# Overexpression and silencing of the cotton *GhABA2* gene reveal its role in salt stress tolerance

**DOI:** 10.3389/fpls.2026.1803231

**Published:** 2026-04-01

**Authors:** Dashuang Cao, Dongliang Guo, Chunyan Gu, Wanwan Fu, Xin Zhang, Wenhong Ma, Jiacong Li, Haixia Jiang, Huixin Zhao

**Affiliations:** 1 Xinjiang Key Laboratory of Special Species Conservation and Regulatory Biology, College of Life Science, Xinjiang Normal University, Urumqi, China; 2 Xinjiang Key Laboratory of Biological Resources and Genetic Engineering, College of Life Science and Technology, Xinjiang University, Urumqi, China

**Keywords:** ABA signalling pathway, cotton, *GhABA2*, salt stress, virus-induced gene silencing

## Abstract

The *GhABA2* gene encodes a short-chain dehydrogenase/reductase involved in abscisic acid (ABA) biosynthesis and plays a crucial role in plant salt stress responses. To explore its function in cotton salt tolerance, we performed bioinformatic analysis, heterologous overexpression in *Arabidopsis*, and virus-induced gene silencing (VIGS) in cotton. Bioinformatic prediction revealed that the promoter region of the *GhABA2* contains multiple cis-acting elements associated with ABA, light, and stress responses. Subcellular localization analysis indicated that GhABA2 was predominantly localized in the cytoplasm. Overexpression of *GhABA2* in *Arabidopsis* significantly enhanced salt tolerance, as manifested by increased germination rates and root elongation. This was accompanied by a reinforced antioxidant system, with elevated activities of catalase (CAT), peroxidase (POD), glutathione reductase (GR), and ascorbate peroxidase (APX), alongside reduced accumulation of malondialdehyde (MDA) and H_2_O_2_. Furthermore, the transgenic lines exhibited higher relative water content (RWC), proline accumulation, and increased endogenous abscisic acid (ABA) levels under salt stress. Consistently, the expression of stress-responsive genes and ABA biosynthesis genes (*AtNCED3*, *AtAAO3*) was upregulated. Conversely, virus-induced gene silencing (VIGS) of *GhABA2* in cotton compromised salt tolerance, characterized by diminished antioxidant enzyme activities (superoxide dismutase SOD, POD, GR, APX), reduced ABA content, and lower RWC and proline levels, but higher MDA and H_2_O_2_ accumulation. Correspondingly, the transcript levels of ROS-related genes and cotton ABA biosynthesis genes (*GhNCED3a*, *GhNCED3c*, *GhAAO3*) were downregulated. These findings demonstrate that *GhABA2* positively regulates salt tolerance by modulating ABA biosynthesis and the antioxidant defense system. These findings elucidate the role of *GhABA2* in modulating cotton salt stress responses and highlight its potential as a genetic target for breeding salt-tolerant cotton varieties.

## 1 Introduction

Soil salinization, or salt stress, denotes the detrimental impact of elevated salt concentrations in the growth environment, such as in soil or irrigation water, on plant growth, development, and physiological metabolism. As a major abiotic stress, it significantly constrains agricultural productivity and threatens global food security (Peng et al., 2025). This issue is particularly acute in the arid northwestern regions of China, where low precipitation, high evaporation rates, and saline groundwater contribute to progressive soil salinization (Gao et al., 2024). Cotton, a key economic crop in China, is predominantly cultivated in Xinjiang. In 2024, Xinjiang produced approximately 5,112 million metric tons of cotton, accounting for 91% of the national total, underscoring its pivotal role in the Chinese cotton industry. However, soil salinization affects nearly 10% of China’s arable land, with northwestern regions being especially vulnerable, which poses a serious threat to cotton production (Ma and Tashpolat, 2023). Notably, saline soils constitute 37.72% of the total cultivated area in Xinjiang’s irrigation zones, posing a substantial challenge to local cotton production (Yang et al., 2016). It has been established that salt stress exacerbates yield loss in cotton by impairing photosynthetic machinery and inducing oxidative damage. Identifying key salt-tolerant genes is therefore central to enhancing cotton salinity tolerance (Zhang et al., 2014).

Abscisic acid (ABA), a key plant stress hormone and physiological regulator, plays a central role in mediating plant salinity tolerance (Wani and S, 2015). Under salt stress, ABA can enhance transpiration efficiency, increase photosynthetic activity, and elevate antioxidant enzyme activity, thereby promoting the growth of rice leaves (Wang X. et al., 2025). During seed germination and the early seedling stage, plants are particularly sensitive to abiotic stresses such as salinity. Pretreatment with ABA has been shown to significantly improve salt tolerance in maize by regulating reactive oxygen species (ROS) homeostasis, organic acid metabolism, and osmotic balance (Sarkar et al., 2023). In tomato, the ABA-deficient mutant notabilis, which carries a loss-of-function mutation in the *NCED1* gene, exhibits increased sensitivity under mild to severe salt stress (Holsteens et al., 2022). Similarly, in rice, the chloroplast-localized ABA biosynthesis gene *OsNCED4* is significantly induced under salt stress and is involved in regulating ROS homeostasis and ABA levels (Xiang et al., 2024). Additionally, overexpression of *HDA15* enhances salt tolerance by upregulating the expression of *NCED3*, thereby promoting ABA accumulation (Truong et al., 2021). In addition, ABA modulates plant stress responses through its interactions with auxin (IAA), gibberellin (GA), and cytokinin (CK) (Yu et al., 2020). In maize, ABA disrupts the polar localization of *ZmPIN1*, leading to altered auxin distribution and consequent inhibition of lateral root formation and development (Lu et al., 2019). In *Arabidopsis*, *ABI4* overexpression enhances the transcription of *NCED6* and *GA2ox7*, increasing the ABA/GA ratio and improving salt tolerance (Shu et al., 2016). Furthermore, ABA suppresses cytokinin biosynthesis via *MYB2*, increasing ABA sensitivity and reducing shoot growth as an adaptive mechanism (Guo and Gan, 2011). Similarly, in tomato, salt stress elevates ABA levels while reducing CK content (Albacete et al., 2008). These studies clearly demonstrate that the ABA play crucial roles in plant adaptation to salt stress.

*ABA2* (Abscisic Acid Deficient 2), first identified and functionally characterized in the model plant *Arabidopsis thaliana*, encodes a short-chain dehydrogenase/reductase that catalyzes a key step in the biosynthesis of ABA (Wang Z. et al., 2025). Extensive studies in *Arabidopsis* have established *ABA2* as a core enzyme in the ABA biosynthesis pathway, serving as a critical regulatory node in plant responses to various abiotic stresses, including drought, high salinity, cold, and nutrient stress (Wang Z. et al., 2025). Mutants such as *aba2-1* exhibit pleiotropic phenotypes including reduced seed dormancy, enhanced transpiration water loss, and altered stress tolerance, underscoring the central role of *ABA2*-mediated ABA synthesis in stress adaptation (González-Guzmán et al., 2002). Orthologs of *ABA2* have been functionally characterized in various plants, revealing both conserved functions and species-specific roles in stress adaptation. For instance, in melon, *CmABA2* encodes a cytoplasmic SDR involved in ABA biosynthesis and has been identified as a key candidate gene associated with fruit ripening and stress resistance, providing a valuable genetic resource for melon improvement (Li et al., 2023). In wheat, the expression level of *TaABA2* is positively correlated with salt tolerance across varieties. Overexpression of *TaABA2* enhances endogenous ABA accumulation, boosts the activity of antioxidant systems, and consequently improves plant survival under salt stress, underscoring its value for breeding salt−tolerant wheat (Yang et al., 2023). In *Arabidopsis*, the *DGK5/PA*–*ABA2* interaction inhibits *ABA2* activity and further compromises plant salt tolerance (Li et al., 2024). Together, these findings demonstrate that *ABA2* as a key rate-limiting enzyme gene in ABA biosynthesis, plays a crucial role in plant adaptation to salt stress.

While the function of *ABA2* in abiotic stress responses has been well-documented in model plants, its orthologs in cotton remain largely uncharacterized. To systematically investigate the function of *GhABA2* in cotton under salt stress, we first conducted a comprehensive bioinformatic analysis of *GhABA2*, including protein structure prediction, domain analysis, promoter cis-element profiling, and phylogenetic reconstruction. We further generated *GhABA2*-overexpressing *Arabidopsis* lines and performed virus-induced gene silencing (VIGS) of *GhABA2* in cotton. Comparative phenotypic and physiological assessments under salt stress were then conducted to elucidate the contribution of *GhABA2* to salt tolerance. Our results provide molecular functional basis into the salt stress response in cotton and highlight *GhABA2* as a promising genetic target for breeding salt-resistant cotton varieties.

## 2 Materials and methods

### 2.1 Plant materials and growth conditions

Wild-type (WT) *Arabidopsis thaliana* seeds were provided by our laboratory. Seeds of the upland cotton (*Gossypium hirsutum*) variety Zhongmian 113 were obtained from Xinjiang Zhongmian Seed Industry Co., Ltd. For *Arabidopsis*, seeds were surface-sterilized by sequential washing with 75% absolute ethanol for 5 min and 20% (v/v) NaClO for 5 min, followed by three rinses with sterile distilled water. The sterilized seeds were sown on half-strength Murashige and Skoog (1/2 MS) solid medium. After stratification at 4 °C for 1-2 days to break dormancy, the plates were transferred to a growth chamber set at 22 ± 1 °C under a 16h light/8h dark photoperiod. Seedlings were grown for 10-14 days before being transplanted into pots containing a mixed substrate of nutrient soil, black soil, and vermiculite (1:3:1, v/v/v) for subsequent cultivation in a controlled environment room. For cotton, seeds were surface-sterilized with ethanol and 25% NaClO solution, then placed between moist sterile filter papers and incubated at 28 °C to promote germination. Germinated seedlings were transplanted into pots filled with a mixed substrate of nutrient soil, black soil, and vermiculite (3:1:1, v/v/v). All plants were subsequently grown in controlled environment rooms under the following conditions: 25 °C (day)/22°C (night), with a 16h light/8h dark photoperiod.

### 2.2 Gene cloning and expression vector construction

The coding sequence (CDS) of *GhABA2* (*Ghi_A13G00706*) was retrieved from the CottonMD database (https://yanglab.hzau.edu.cn/CottonMD). Gene-specific primers (**Supplementary Table 1**) were designed, and the full-length CDS was amplified from cotton cDNA via polymerase chain reaction (PCR). The purified PCR product was sequenced for verification. The confirmed *GhABA2* CDS was subsequently cloned into the pCAMBIA-2300-GFP vector, generating the recombinant overexpression plasmid pCAMBIA-2300-GFP-*GhABA2*. For VIGS, a 400 bp specific fragment of *GhABA2* was amplified and cloned into the TRV2 vector to construct TRV2:*GhABA2*. All constructed plasmids were verified by sequencing and subsequently introduced into *Agrobacterium tumefaciens* strain GV3101 competent cells for plant transformation.

### 2.3 Bioinformatics analysis

Bioinformatic analysis was performed on the GhABA2 protein. Its physicochemical properties were predicted using the ProtParam tool. Phosphorylation sites were analyzed with NetPhos. A hydrophobicity plot was generated using the Kyte & Doolittle algorithm. Subcellular localization was predicted by WoLF PSORT. Secondary and tertiary structures were predicted using SOPMA and SWISS-MODEL, respectively. A phylogenetic tree was constructed via the neighbor-joining method in MEGA software. Cis-regulatory elements in the promoter region were analyzed using the PlantCARE database. The online tools utilized for these analyzes are summarized in **Supplementary Table 1**.

### 2.4 Subcellular localization

The recombinant plasmid pCAMBIA-2300-GFP-*GhABA2* was introduced into *Agrobacterium tumefaciens* strain GV3101. The transformed bacteria were cultured in LB liquid medium supplemented with 30 ug/mL kanamycin and 25 ug/mL rifampicin for 24 h. The bacterial cultures were centrifuged, and the pellets were resuspended in an infiltration buffer containing 120µM acetosyringone (AS), 10mM MES and 10mM MgCl_2_. The optical density at 600nm (OD600) of the bacterial suspension was adjusted to approximately 0.6, followed by incubation in darkness for 3h. The suspension was then infiltrated into the abaxial side of leaves from 4-week-old *Nicotiana benthamiana* using a needleless syringe. After incubation under low-light conditions for 48h, epidermal cells of the infiltrated leaf areas were observed using a confocal laser scanning microscope.

### 2.5 NaCl and exogenous ABA treatments

Cotton seedlings at the three−leaf−one−heart stage were gently rinsed with deionized water to remove any adhering substrate from the roots. For salt−stress treatment, seedlings were transferred to containers filled with 100mL of 300mM NaCl solution (treatment group) or an equal volume of deionized water (control group) and subjected to hydroponic culture. Leaf samples were collected at 0, 3, 6, and 12 h after the onset of treatment. ABA powder was initially dissolved in a small volume of anhydrous ethanol to prepare a 10 mM stock solution, which was stored at −20 °C in the dark until use. Prior to treatment, the stock solution was diluted with deionized water to a final working concentration of 100 μM ABA. To ensure consistent solvent conditions, the control solution was prepared by adding an equivalent volume of anhydrous ethanol to deionized water, resulting in a final ethanol concentration identical to that of the ABA treatment solution (≤0.1%, v/v). Uniformly developed seedlings at the same growth stage were selected for foliar spraying at 10:00 a.m. Each plant in the treatment group received 150 μL of 100 μM ABA solution to activate ABA-responsive signaling, whereas control plants were sprayed with an equal volume of deionized water containing the same concentration of ethanol. Leaves were harvested at 0, 3, 6, 12, and 24 h post-spraying. The expression level of *GhABA2* was quantified by quantitative reverse−transcription PCR (qRT−PCR) with gene−specific primers.

### 2.6 Generation and identification of transgenic plants

To generate *Arabidopsis* overexpression lines, the recombinant plasmid pCAMBIA-GFP-2300-*GhABA2* was introduced into GV3101. The transformed bacteria were cultured in LB liquid medium containing 30 mg/L kanamycin and 25 mg/L rifampicin for 24 h. After centrifugation, the bacterial pellet was resuspended in an infiltration buffer (containing 5% (w/v) sucrose, 0.01% (w/v) surfactant Silwet-77, and 0.21% (w/v) MS powder). The Optical Density at 600 nm (OD600) was adjusted to approximately 0.7. *Arabidopsis* Col-0 plants were transformed using the *Agrobacterium*-mediated floral dip method. T1 seeds were screened on 1/2 MS medium containing 50 mg/L kanamycin. Positive transgenic plants were identified by genomic PCR and the qRT−PCR. To ensure the accuracy of target gene expression analysis, the reference gene was first systematically validated in this study. The amplification efficiencies of *GhABA2* and *Actin2* were determined to be 102.56% and 101.07%, respectively, by qRT-PCR, both falling within the acceptable range (90%–110%) for qRT-PCR experiments (**Supplementary Figures S1A, B**). To further verify the reliability of *Actin2* as an internal control, its expression stability was assessed under salt treatment. The results showed no significant fluctuation in *Actin2* expression levels before and after salt treatment, indicating its robust stability and suitability for gene expression normalization under different experimental conditions (**Supplementary Figure S5**). Based on this validation, *Actin2* was used as the reference gene to precisely quantify the relative expression levels of *GhABA2* in transgenic cotton plants. Homozygous T3 lines exhibiting significantly elevated *GhABA2* expression were subsequently selected for further functional studies.

To generate VIGS cotton plants, the recombinant plasmid TRV2:*GhABA2*, along with the control plasmids TRV1, TRV2:00, and TRV2:*GhCLA*, were separately introduced into GV3101. The corresponding bacterial cultures were grown under the same conditions, centrifuged, and the pellets were resuspended in an infiltration buffer (containing 10mM MgCl_2_, 10mM MES, and 150µM AS). The OD600 of each suspension was adjusted to approximately 0.8. The bacterial suspension carrying *TRV1* was mixed separately at a 1:1 ratio with suspensions containing TRV2:*GhABA2*, TRV2:00 and TRV2:*GhCLA*, respectively. After incubation in darkness for 3 h, the mixtures were infiltrated into the cotyledons of two week-old cotton seedlings. The plants were kept in darkness for 24 h before being returned to normal growth conditions. The amplification efficiencies of the target gene *GhABA2* and the reference gene *GhHis3* were determined to be 104.80% and 105%, respectively, by qRT-PCR, both falling within the acceptable range (90%–110%) for qRT-PCR experiments (**Supplementary Figures S1C, D**). To assess the expression stability of *GhHis3* under salt treatment, its transcript levels were evaluated before and after salt exposure. The results showed no significant fluctuations, indicating that *GhHis3* is stably expressed under salt conditions and is suitable for subsequent normalization of gene expression analysis (**Supplementary Figure S5**). At the true-leaf stage of cotton plants, the silencing efficiency of *GhABA2* was examined by qRT-PCR using *GhHis3* as the internal reference, with TRV2:*GhCLA* plants serving as the positive control for the VIGS system. Based on the expression analysis, plants exhibiting significantly downregulated *GhABA2* expression were selected for subsequent functional studies.

### 2.7 Phenotypic analysis of transgenic plants under salt stress

Germination Assay: Surface-sterilized seeds of transgenic and WT *Arabidopsis* were sown on 1/2 MS solid medium supplemented with 0, 100, 150, or 200mM NaCl, following the standard culture method described in section 2.1. Germination rates were recorded after 10 days. Root Elongation Assay: Surface-sterilized seeds of transgenic and wild-type *Arabidopsis* were germinated on 1/2 MS solid medium for 4 days. Uniformly germinated seedlings were then transferred to vertical plates containing 1/2 MS medium with 0, 100, 150, or 200mM NaCl. The primary root length was measured after 4 days of vertical growth (Zhang et al., 2024). Whole Plant Salt Tolerance Assay in *Arabidopsis*: Fourteen day-old *Arabidopsis* seedlings were subjected to salt stress by irrigation with a 200mM NaCl solution every 3 days for a total of 18 days (Song et al., 2023; Sun et al., 2025). Phenotypes were photographed at the end of the treatment period. Salt Stress Treatment in Cotton: At the true-leaf stage, TRV2:00 and TRV2:*GhABA2* cotton plants were transferred to a hydroponic system containing distilled water for a 2-day acclimation period. Salt stress was then applied by gradually increasing the NaCl concentration in a stepwise manner from 50 mM to 100, 200, and finally 300 mM over an 8-hour period. Thereafter, phenotypic changes were observed and recorded at various time points during continuous treatment with 300 mM NaCl (Wang et al., 2017; Guo et al., 2019).

To investigate the functional specificity of *GhABA2* in ABA-mediated salt tolerance, a phenotypic rescue experiment was performed. Uniformly grown *GhABA2*-silenced (TRV2:*GhABA2*) and control (TRV2:00) plants were first subjected to salt stress. Immediately after stress treatment, both groups were foliar-sprayed with a 100 µM ABA solution at a volume of 3 mL per plant until runoff (ensuring uniform coverage without dripping). Phenotypic changes were observed and documented at 0 and 6 hours post-treatment, with photographs taken at each time point. At least three individual plants were examined per time point. At 6 hours post-application, the phenotypic recovery of the silenced lines was assessed and compared with that of the control plants (Mahmoud et al., 2020; Hu et al., 2022).

### 2.8 Measurement of physiological and biochemical parameters

To evaluate the ROS-mediated oxidative stress response in *GhABA2*-overexpressing *Arabidopsis* and *GhABA2*-silenced cotton plants under salt stress, leaf samples from *Arabidopsis* were collected at 3 days of salt treatment and under normal control conditions. For cotton, true leaves were harvested after 6 h of salt treatment and from untreated control plants. According to the manufacturers’ instructions of commercial assay kits, the following parameters were measured: POD, SOD,CAT,GR,APX and activities; MDA, Pro and H_2_O_2_ contents (Solarbio, Beijing, China); and RWC. To further elucidate the molecular basis underlying *GhABA2*-mediated salt tolerance, we examined the transcript levels of key genes involved in the ABA biosynthesis pathway and ROS homeostasis in transgenic *Arabidopsis* and cotton VIGS plants before and after salt stress using qRT-PCR (**Supplementary Table** List of primers) (Endo et al., 2008; Cai et al., 2021; Wang et al., 2023; Xia et al., 2023).

### 2.9 Measurement of endogenous ABA levels

A amount of plant leaves (typically 1 g, with a minimum of 50 mg) was weighed and homogenized in an ice bath with PBS (0.01 mol/L, pH 7.2–7.4) at a ratio of 1:9 (w/v), corresponding to a 10% homogenate concentration. The homogenate was centrifuged at 2000–3000 rpm for 20 min, and then the supernatant was collected for subsequent analysis. ABA content was detected by an enzyme-linked immunosorbent assay (ELISA) kit (Jiangsu Jingmei Biological Technology Co., Ltd., Yancheng, China), following the manufacturer’s instructions. Detailed steps are described in the **Supplementary Information**.

### 2.10 RNA extraction and qRT-PCR analysis

Total RNA was extracted from leaves of transgenic *Arabidopsis*, TRV2:*GhABA2* cotton and control plants using the Plant Polysaccharide & Polyphenol RNA Kit (FOREGENE, Chengdu, China), following the manufacturer’s protocol. RNA quality was verified by agarose gel electrophoresis, and concentration was determined using a NanoDrop 2000 spectrophotometer (Thermo Fisher Scientific, USA). First-strand cDNA was synthesized with the FOREGENE Master Premix RT Easy™ II Kit. qRT-PCR was performed using Real Time PCR Easy™-SYBR Green I reagents on a CFX96 Touch Real-Time PCR Detection System (Bio-Rad, USA). The thermal cycling conditions consisted of an initial denaturation at 95 °C for 30 seconds, followed by 40 cycles of 95 °C for 5 seconds and 60 °C for 30 seconds. Relative expression levels were calculated using the 2^−ΔΔCT^ method.

### 2.11 Data analysis

Statistical analysis was performed using GraphPad Prism 9 software (San Diego, CA, USA). For cotton gene expression data, statistical significance relative to reference samples was assessed using Student’s *t*-test. For physiological measurements in cotton, after confirming normality by the Shapiro–Wilk (SW) test, two-way ANOVA followed by Sidak multiple comparisons test was applied. For *Arabidopsis* data, including germination rate, root length, and physiological parameters, normality was first verified using the Shapiro–Wilk test, followed by two-way ANOVA and Tukey multiple comparisons test. All graphs were generated with GraphPad Prism. All experiments included three biological replicates with three technical replicates each. Error bars are uniformly presented as mean ± standard deviation (SD).

## 3 Results

### 3.1 Molecular cloning and bioinformatic characterization of *GhABA2*

Through PCR and sequencing analysis, an 849 bp CDS fragment of *GhABA2* was obtained and the sequence was confirmed to be complete and accurate, matching the reference *GhABA2* CDS. These results confirm the successful cloning of the full-length *GhABA2* CDS for further functional characterization.

**Figure 1:**
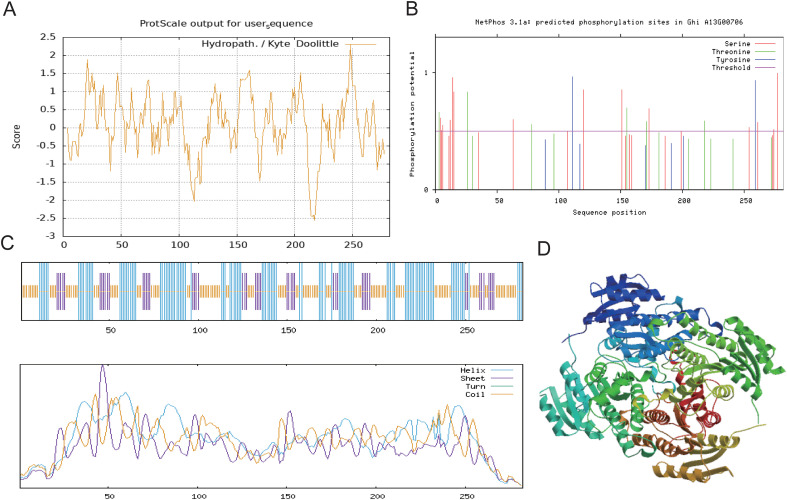
Protein analysis of *GhABA2*. **(A)** Hydrophilicity/hydrophobicity analysis; **(B)** Phosphorylation site prediction; **(C)** Secondary structure prediction; **(D)** Tertiary structure prediction.

Bioinformatic analysis revealed that the GhABA2 protein consists of 282 amino acids, with a molecular formula of C_1317_H_2088_N_374_O_405_S_12_ and a molecular weight of approximately 30.03 kDa. The theoretical isoelectric point (pI) was predicted to be 6.1. The protein contains 23 basic (Arg+Lys) and 28 acidic (Asp+Glu) residues, which may suggest a potential capacity for ionic interactions. The instability index was computed as 21.25, the protein is predicted to be stable. The aliphatic index was 93.62, indicating a high proportion of aliphatic amino acids that could contribute to thermal stability. The grand average of hydropathicity (GRAVY) was 0.072. Subcellular localization predictions supported a possible cytoplasmic localization. Hydrophobicity analysis suggested that the C-terminal region is predominantly hydrophobic, the N-terminal is hydrophilic, and the central region exhibits alternating hydrophobicity and hydrophilicity. Overall, the protein is hydrophilic, with one prominent hydrophilic peak exceeding a value of 2 (**Figure 1A**). Multiple potential phosphorylation sites were predicted with high confidence (scores ≈ 1) (**Figure 1B**), implying regulatory potential through phosphorylation. Secondary structure prediction indicated that α-helices (45.73%), random coils (28.25%), extended strands (17.07%), and β-turns (8.94%) constitute the protein (**Figure 1C**). The tertiary structure was modeled using 7o6p.1.A as a template (sequence identity: 51%) (**Figure 1D**).

**Figure 2:**
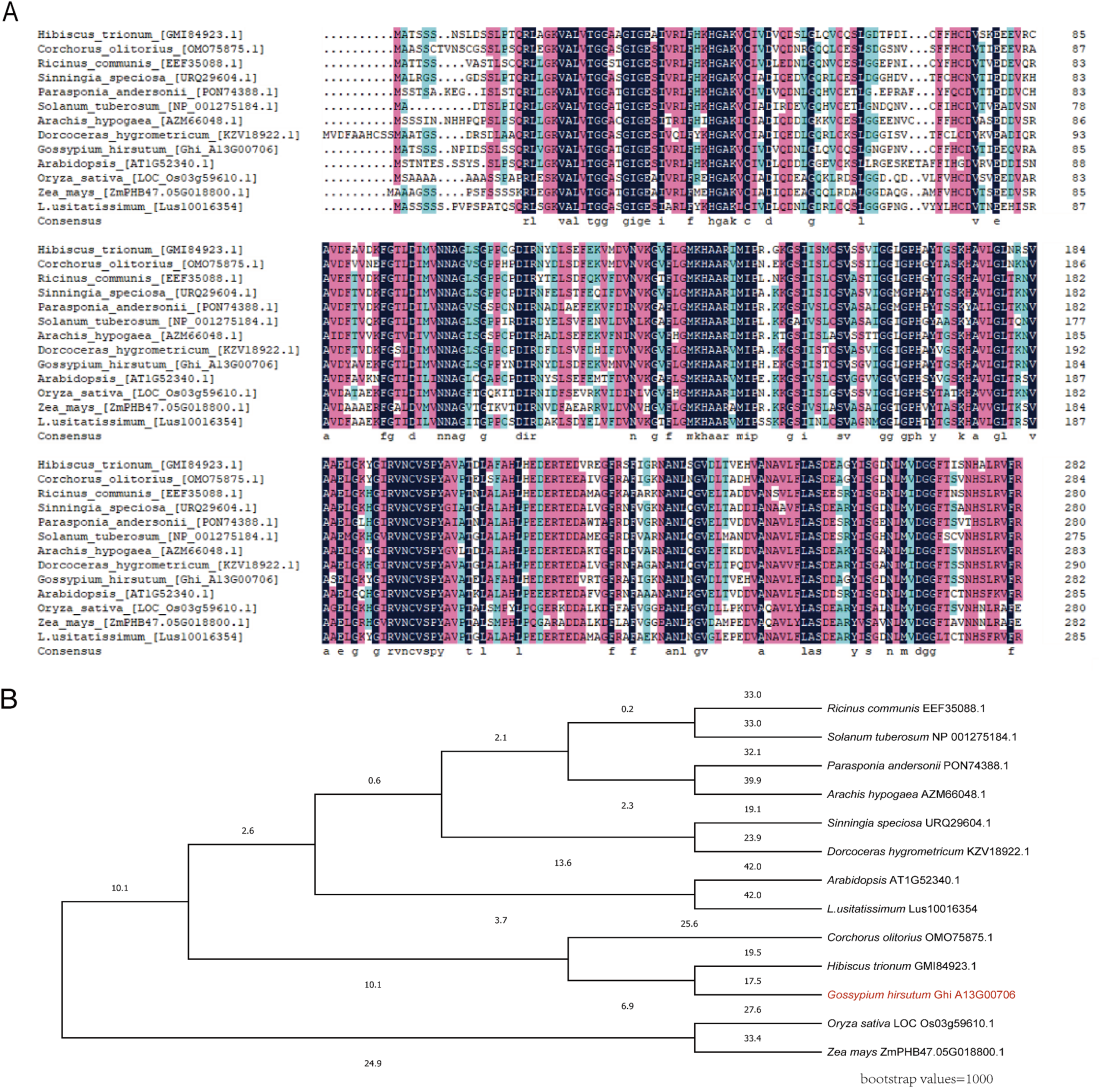
Multiple sequence alignment and phylogenetic analysis of *GhABA2* across different species. **(A)** Multiple sequence alignment among various species; **(B)** Phylogenetic tree analysis.

Multiple sequence alignment demonstrated that GhABA2 contains regions highly conserved among ABA2 homologs from diverse plant species, including *Arabidopsis thaliana*, *Zea mays*, *Oryza sativa*, *Linum usitatissimum*, *Citrullus lanatus*, *Corchorus olitorius*, *Streptocarpus spp*, *Sinningia speciosa*, *Arachis hypogaea*, several tropical trees, *Solanum tuberosum*, and *Ricinus communis* (**Figure 2A**), underscoring the functional conservation of this gene. Phylogenetic analysis revealed that GhABA2 is most closely related to the ABA2 protein from *Citrullus lanatus* (**Figure 2B**), suggesting shared functional and regulatory mechanisms.

Analysis of the 2,000 bp promoter region upstream of the *GhABA2* transcription start site predicted canonical core promoter elements (e.g., TATA-box and CAAT-box). In addition, numerous cis-acting elements associated with hormone response and stress signaling were detected, including abscisic acid response elements (ABRE), gibberellin response elements (GARE-motif), jasmonic acid response elements (CGGTA-motif, TGACG-motif), anaerobic response elements (ARE), and light-responsive elements (G-Box, GT1-motif, Box 4) (**Supplementary Table 2**). The prevalence of these regulatory motifs suggests that *GhABA2* expression is likely modulated by multiple hormones and environmental stimuli, implicating its role in abiotic stress adaptation and hormonal signaling.

### 3.2 Subcellular localization analysis

Subcellular localization analysis revealed that the *GhABA2* was primarily localized in the cytoplasm. Following Agrobacterium-mediated transient expression in Nicotiana benthamiana leaves, confocal laser scanning microscopy performed 48h post-infiltration showed a distinct distribution pattern of the fluorescent signal for the *GhABA2* fusion protein, in contrast to the diffuse signal in the empty vector control (**Supplementary Figure S2**). This result is consistent with the earlier bioinformatic prediction, confirming the cytoplasmic localization of *GhABA2*.

### 3.3 Expression analysis of *GhABA2* under salt stress and exogenous ABA application

**Figure 3:**
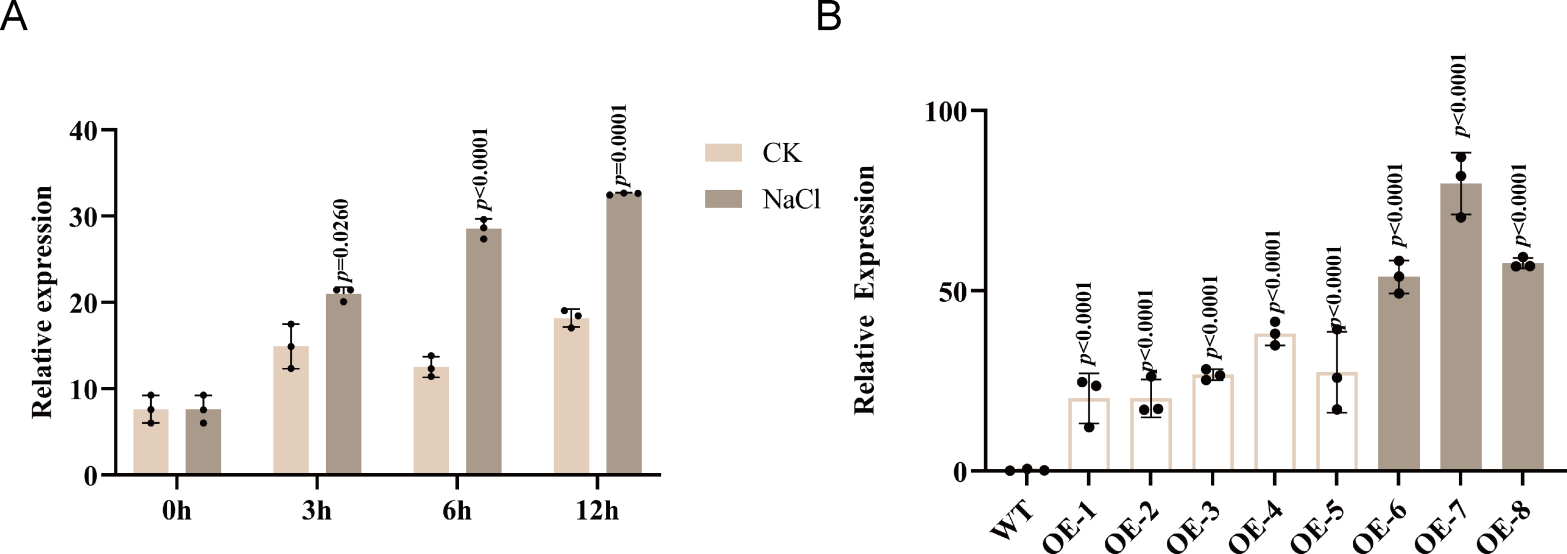
Expression levels of *GhABA2* in cotton under salt stress and transgenic *Arabidopsis*. **(A)** Expression levels of *GhABA2* in cotton plants under salt stress and control conditions at different time points; **(B)** Expression levels of transgenic *Arabidopsis* lines. All data are presented as the mean ± standard deviation (SD).

To examine the response of *GhABA2* to salt stress, its transcript abundance was quantified using qRT-PCR in cotton plants grown hydroponically and treated with either distilled water (control) or 300 mM NaCl. Salt stress treatment significantly up-regulated the expression of *GhABA2* compared to the control (**Figure 3A**), indicating its involvement in the cotton salt stress response.

Motif analysis of the *GhABA2* promoter region revealed the presence of ABA-responsive regulatory elements (**Supplementary Table 3**); however, promoter sequence prediction alone cannot directly confirm its hormonal responsiveness. To clarify the ABA-responsive characteristics of *GhABA2*, an exogenous ABA treatment experiment was conducted. Using untreated samples as controls, gene expression levels were examined at different time points after treatment. The results showed that, compared with the control, exogenous ABA treatment significantly upregulated *GhABA2* expression, with peak levels observed at 6 h and 12 h post-treatment. Although expression slightly decreased at 24 h, it remained significantly higher than that at 0 h (initial state) (**Supplementary Figure S3**). These findings confirm that exogenous ABA application induces the upregulation of *GhABA2*, thereby demonstrating its ABA-responsive nature.

### 3.4 Overexpression of *GhABA2* enhances salt tolerance in *Arabidopsis*

To functionally characterize *GhABA2*, transgenic *Arabidopsis* lines overexpressing this gene were generated. A total of eight independent lines were validated by PCR and qRT-PCR. Among these, three lines (OE6, OE7, and OE8) exhibiting the highest *GhABA2* expression levels (**Figure 3B**) were selected for subsequent phenotypic analysis. Analysis of the three high-expression lines revealed that overexpression of *GhABA2* significantly reduced the 1,000-seed weight compared to the wild type (**Supplementary Figure S4**).

**Figure 4:**
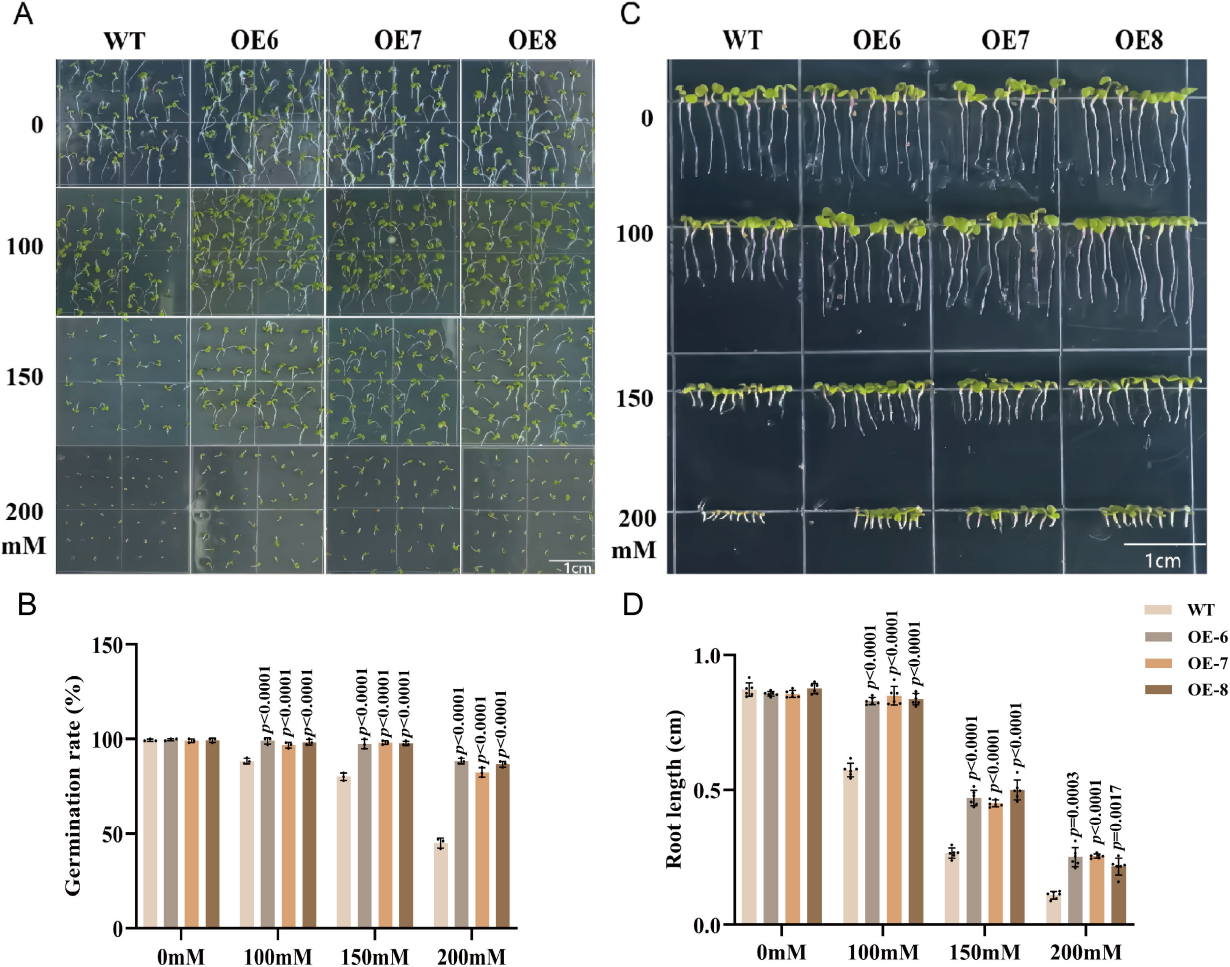
Overexpression of *GhABA2* enhances salt tolerance during germination in *Arabidopsis*. **(A)**Representative germination phenotypes; **(B)** Germination rate; **(C)** Root growth phenotypes; **(D)** Root length. All data are presented as the mean ± SD.

Under salt stress conditions during seed germination, the germination rate of WT seeds decreased markedly with increasing NaCl concentrations. In contrast, the OE lines maintained significantly higher germination rates (**Figures 4A, B**), indicating reduced sensitivity to salt stress at the germination stage. Consistent with the germination phenotypes, root growth assays revealed that although NaCl suppressed root elongation in all genotypes, the OE lines developed significantly longer roots than the WT under each salinity treatment. Notably, at 200mM NaCl, root growth was nearly abolished in WT plants, whereas the OE lines retained considerable root elongation capacity (**Figures 4C, D**).

**Figure 5:**
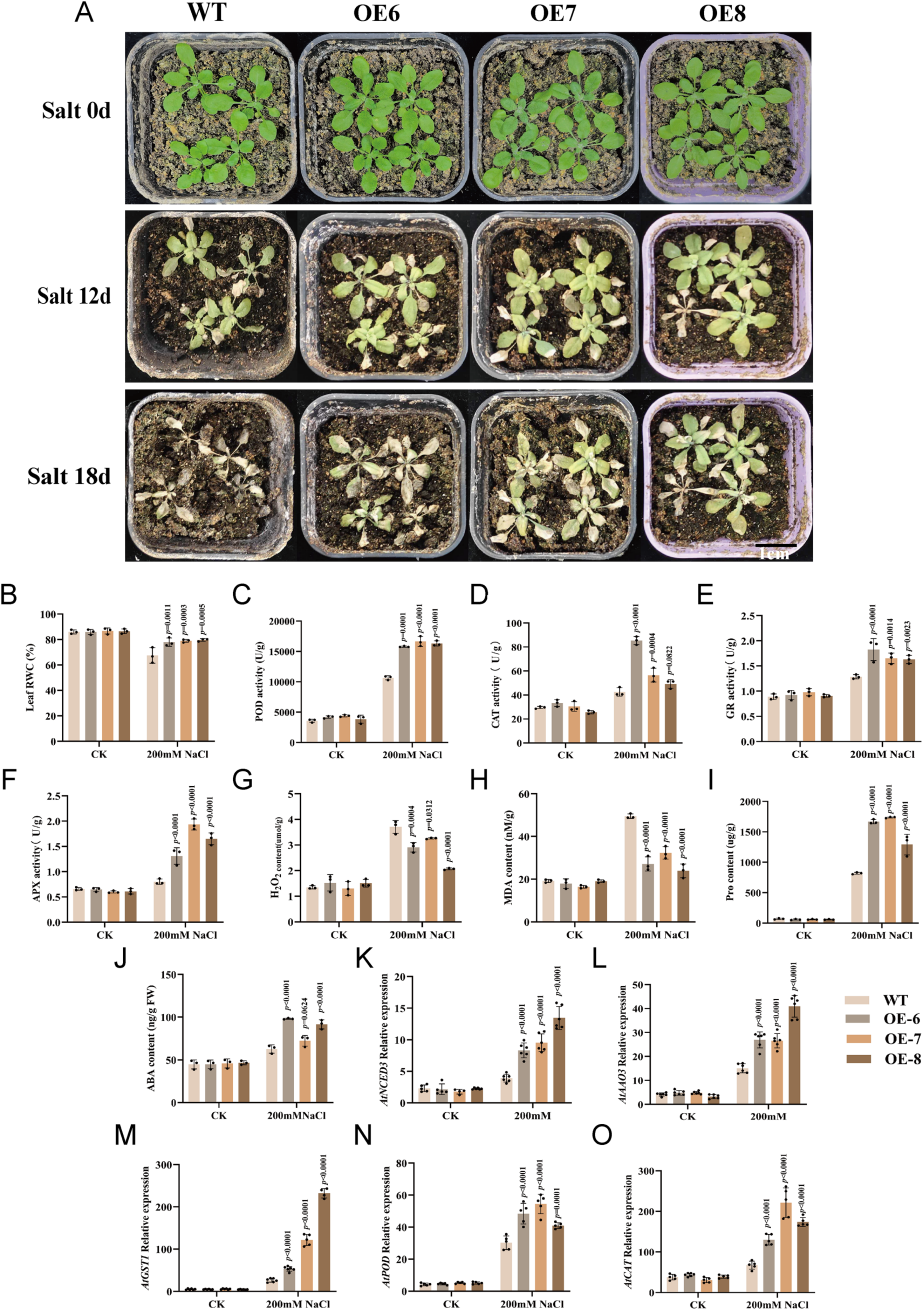
Overexpression of *GhABA2* enhances salt stress tolerance at the seedling stage in *Arabidopsis*. **(A)** Phenotypic comparison of WT and transgenic plants under salt stress; **(B)** RWC; **(C)** POD activity; **(D)** CAT activity; **(E)** GR activity; **(F)** APX activity; **(G)** H_2_O_2_ content; **(H)** MDA content; **(I)** Pro content; **(J)** ABA content; **(K)** Relative expression level of *AtNCED3*; **(L-O)** Relative expression level of *AtAAO3* **(L)**, *AtPOD* **(M)**, *AtGST1* **(N)**, and *AtCAT* **(O)**. All data are presented as the mean ± SD.

To evaluate salt tolerance at the seedling stage, 14-day-old WT and OE plants were subjected to 200 mM NaCl treatment. At the initial stage of treatment, no obvious phenotypic differences were observed between the genotypes. However, after 12 days of treatment, WT plants began to exhibit wilting and leaf necrosis, whereas OE plants showed only mild symptoms of salt stress with limited leaf necrosis. By 18 days post-treatment, WT seedlings displayed severe salt injury, with the majority of individuals succumbing to death; in contrast, the majority of OE plants maintained turgid leaves and exhibited significantly higher survival rates, with only occasional plant death observed (**Figure 5A**).

To further elucidate the physiological mechanisms underlying enhanced salt tolerance, key stress-related biochemical parameters were measured and compared between overexpression lines under normal conditions and those subjected to salt stress. Under normal growth conditions, no significant differences were observed between WT and OE plants in terms of POD, CAT, GR, and APX activities, as well as RWC, MDA, Pro, and endogenous ABA levels. However, under salt stress, OE plants exhibited significantly higher activities of POD, CAT, GR, and APX (**Figures 5C–F**), along with elevated RWC, Pro, and ABA contents (**Figures 5B, I–J**). Consistently, the accumulation of MDA and H_2_O_2_ was markedly lower in OE plants than in WT (**Figures 5G, H**), indicating reduced oxidative damage. To further investigate the molecular mechanism underlying *GhABA2*-mediated salt tolerance, we examined the expression of genes involved in ABA biosynthesis and ROS signaling pathways using qRT-PCR. The results showed that under salt stress, the transcript levels of key ABA biosynthesis genes (*AtNCED3*, *AtAAO3*) and ROS-scavenging genes (*AtPOD*, *AtGST1*, *AtCAT*) were significantly higher in OE plants than in WT plants (**Figures 5K–O**). Taken together, these findings demonstrate that heterologous overexpression of *GhABA2* enhances salt tolerance in *Arabidopsis* by coordinately modulating ROS homeostasis and ABA biosynthesis pathways.

### 3.5 Silencing of *GhABA2* impairs salt tolerance in cotton

**Figure 6:**
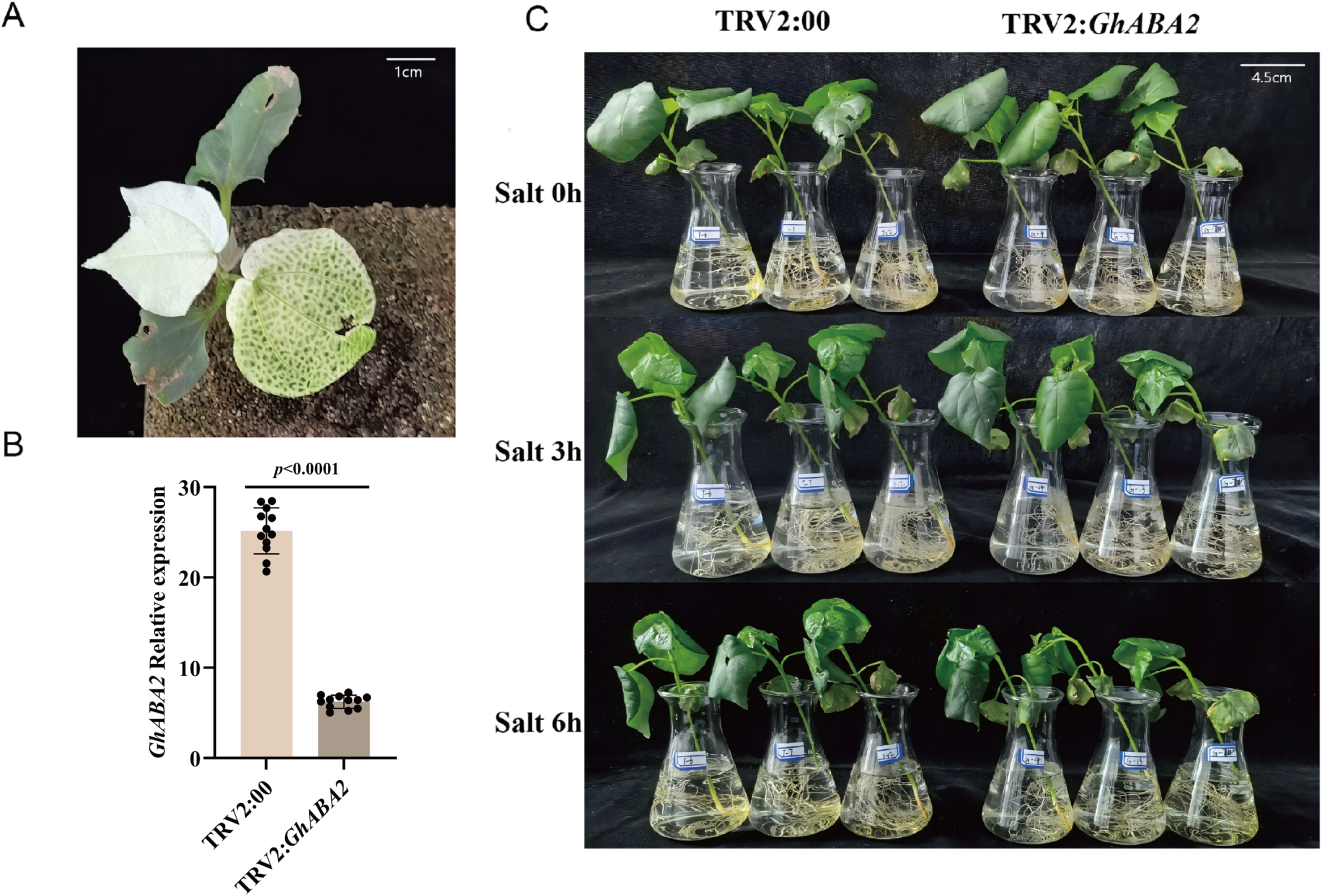
Silencing of GhABA2 reduces cotton tolerance to salt stress. **(A)** Albino marker plants; **(B)** Relative expression of GhABA2 in TRV2:00 and TRV2: GhABA2 plants; **(C)** Phenotype of TRV2:00 and TRV2:GhABA2 plants under salt treatment. All data are presented as the mean ± SD.

To further investigate the role of *GhABA2* in salt stress response, 30 cotton seedlings each were inoculate with TRV2:*GhABA2* and TRV2:00, respectively. When the positive control plants (TRV2:*GhCLA*) exhibited the expected albino phenotype, effective silencing was confirmed (**Figure 6A**). The expression levels of the target gene were detected by qPCR and compared between the TRV2:00 and TRV2:*GhABA2* groups. Plants with comparable silencing efficiencies between the TRV2:00 and TRV2:*GhABA2* groups were selected for subsequent experiments. A total of 12 plants with uniform growth were selected for salt treatment. (**Figure 6B**). From these, three plants were randomly chosen for subsequent phenotypic photography, and the remaining plants were used for sampling and physiological index analysis. In addition, To assess the specificity of the VIGS targeting *GhABA2*, the transcriptional levels of four *GhABA2* paralogous genes in cotton were systematically examined by qRT-PCR. First, the amplification efficiency and specificity of the primers were validated. All primers exhibited amplification efficiencies within the optimal range of 90%–110%, and both melting curve analysis and agarose gel electrophoresis confirmed the presence of single, specific amplification products, demonstrating high primer specificity. Subsequently, the expression levels of these four paralogs were compared between TRV2:*GhABA2* silencing plants and control TRV2:00 plants. No significant differences in expression were detected between the silenced and control plants for any of the paralogous genes, thereby ruling out potential off-target effects. Collectively, these results demonstrate that the VIGS treatment employed in this experiment specifically and effectively silences the target gene *GhABA2* (**Supplementary Figure S6**). At the three true-leaf stage, both TRV2:00 and TRV2:*GhABA2* plants were treated with 300 mM NaCl. While no obvious phenotypic differences were observed at the beginning of treatment (0 h), TRV2:*GhABA2* plants displayed mild wilting after 3 h. Following 6 h of salt stress, the wilting phenotype in silenced plants became more pronounced compared with the control (**Figure 6C**), indicating that silencing of *GhABA2* leads to reduced salt tolerance.

To further validate the specific role of *GhABA2* in ABA-mediated salt tolerance, a phenotypic rescue experiment was conducted. Following exposure to salt stress, *GhABA2*-silenced (TRV2:*GhABA2*) and control (TRV2:00) plants were foliar-sprayed with a 100 µM ABA solution. Notably, the exogenous ABA treatment progressively alleviated the salt-sensitive phenotype of the *GhABA2*-silenced plants. Within 6 hours post-application, the silenced plants recovered to a phenotype comparable to that of the control plants (**Supplementary Figure S7**).

**Figure 7:**
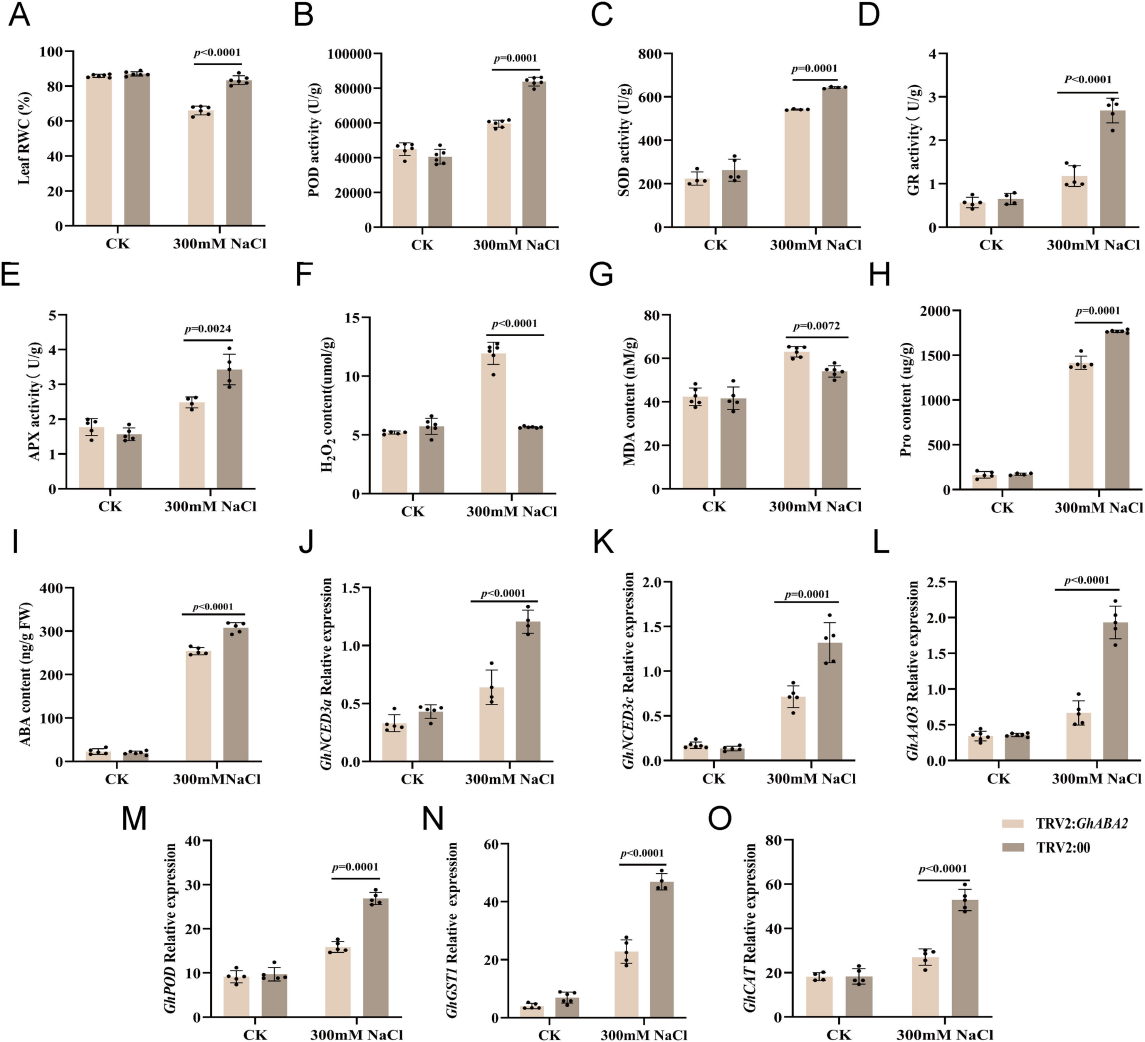
Physiological analysis of *GhABA2*-silenced cotton plants under salt stress. **(A)** RWC; **(B)** POD activity; **(C)** SOD activity; **(D)** GR activity; **(E)** APX activity; **(F)** H_2_O_2_ content; **(G)** MDA content; **(H)** Pro content; **(I)** ABA content; **(J)** Relative expression level of *GhNCED3a*; **(K)** Relative expression level of *GhNCED3c*; **(L-O)** Relative expression level of *GhAAO3* **(L)**, *GhPOD* **(M)**, *GhGST1* **(N)**, and *GhCAT* **(O)** in TRV2:00 and TRV2:*GhABA2* plants. All data are presented as the mean ± SD.

To investigate the physiological basis of this phenotype, key stress-related physiological parameters were measured under both normal conditions and salt stress. Under normal growth conditions, no significant differences were observed between TRV2:*GhABA2* and TRV2:00 plants in terms of POD, SOD, GR, and APX activities, as well as ABA content, RWC, MDA, Pro, and H_2_O_2_ levels. However, under salt stress, the TRV2:*GhABA2* lines exhibited marked physiological and molecular alterations compared to the controls. First, the activities of antioxidant enzymes, including POD, SOD, GR, and APX, were significantly reduced (**Figures 7B–E**). Second, osmotic adjustment capacity was impaired, as evidenced by decreased ABA, RWC, and Pro contents (**Figures 7A, H, I**). Concomitantly, oxidative damage was exacerbated, with significantly higher accumulation of MDA and H_2_O_2_ (**Figures 7F, G**). To further elucidate the molecular basis of *GhABA2*-mediated salt tolerance in cotton, we examined the expression of genes involved in ABA biosynthesis and ROS scavenging using qRT-PCR. The results showed that under salt stress, the transcript levels of ABA biosynthesis pathway genes (*GhNCED3a*, *GhNCED3c*, *GhAAO3*) and ROS-scavenging genes (*GhPOD*, *GhGST1*, *GhCAT*) were significantly downregulated in TRV2:*GhABA2* plants compared to control plants (**Figures 7J–O**). Taken together, these findings suggest that silencing of *GhABA2* compromises salt tolerance in cotton, likely through impaired ABA signaling, diminished ROS scavenging capacity, and suppressed proline-mediated osmotic adjustment.

## 4 Discussion

ABA serves as a central regulator of plant adaptation to abiotic stress (Zhang et al., 2025). Under optimal conditions, ABA is maintained at a low baseline level that permits maximal growth, whereas its content rises rapidly upon exposure to abiotic stress to mediate plant acclimation (Yu et al., 2021; Deng et al., 2022; You et al., 2023). It orchestrates a spectrum of responses, including accumulation of osmolytes and activation of the antioxidant system (Fujita et al., 2013). ABA biosynthesis is tightly regulated, with the short-chain dehydrogenase/reductase *ABA2* catalyzing a key cytoplasmic step: the conversion of xanthoxin to ABA-aldehyde (Seo et al., 2000; Dong et al., 2015). Although the core ABA biosynthetic pathway is conserved, its regulation and integration with stress signaling networks exhibit species-specific characteristics. Therefore, this study elucidates the role of *GhABA2* in cotton, demonstrating that it fine-tunes endogenous ABA levels to coordinate tolerance mechanisms under salt stress.

Salt stress triggers the overaccumulation of reactive oxygen species (ROS), which is a primary cause of oxidative damage in plants. Stress signals can induce ROS burst through various pathways, such as the activation of plasma membrane NADPH oxidases, leading to membrane lipid peroxidation and cellular damage (Zhu et al., 2025). To counteract oxidative stress, plants have evolved a sophisticated antioxidant defense system. Salt-tolerant plants typically exhibit enhanced ROS scavenging capacity, which relies not only on the coordinated upregulation of antioxidant enzymes (e.g., SOD, POD, CAT) but also on the maintenance of non-enzymatic antioxidants such as ascorbate and glutathione (Cui et al., 2025). APX and GR are key enzymes in the ascorbate-glutathione (AsA-GSH) cycle, functioning synergistically to scavenge excess H_2_O_2_. APX catalyzes the reduction of H_2_O_2_ to H_2_O using ascorbic acid (AsA) as an electron donor, generating monodehydroascorbate (MDHA). Meanwhile, GR regenerates reduced glutathione (GSH) from oxidized glutathione (GSSG), thereby maintaining the cellular pool of reduced antioxidants and ensuring the sustained operation of the AsA-GSH cycle (Lu et al., 2007). Concurrently, osmotic and ionic imbalances caused by salt stress rapidly induce the accumulation of ABA. ABA further regulates downstream responses, including synthesis of osmoregulatory substances such as proline (Waheed et al., 2024) and the mobilization of the antioxidant system. In this process, POD, while scavenging H_2_O_2_, may also participate in cell wall cross-linking to enhance the physical barrier (Wu et al., 2017); CAT activity displays spatiotemporal specificity, and its long-term upregulation is crucial for clearing large amounts of H_2_O_2_ generated in peroxisomes through metabolism such as photorespiration (Mhamdi et al., 2010; Fransen and Lismont, 2024). Together, these components form a multi-layered defense network spanning ROS conversion, H_2_O_2_ clearance, and detoxification of secondary toxic compounds. This study focuses on the potential role of *GhABA2* in *Arabidopsis* and cotton under salt stress. Our results demonstrated that overexpression of *GhABA2* may be involved in regulating endogenous ABA biosynthesis and is associated with the activation of antioxidant and osmoregulatory pathways. Under salt stress, compared with WT plants, *GhABA2*-overexpressing lines showed partial alleviation of inhibition in seed germination and root elongation. This improved salt tolerance phenotype was closely associated with enhanced ROS scavenging capacity, as evidenced by significantly increased activities of CAT, POD, GR, and APX, along with reduced H_2_O_2_ accumulation and upregulated transcript levels of key ABA biosynthesis genes (*AtNCED3*, *AtAAO3*) and multiple ROS-related genes. Furthermore, OE lines displayed reduced membrane lipid peroxidation (as indicated by lower MDA content), increased proline accumulation, and higher RWC, suggesting enhanced osmotic adjustment capacity (**Figures 5B–O**). In contrast, silencing of GhABA2 in cotton resulted in pronounced physiological disturbances under salt stress, including significantly decreased activities of SOD, POD, GR, and APX, reduced endogenous ABA and proline content, elevated H_2_O_2_ accumulation, and increased MDA levels. Consistently, the expression of key ABA biosynthesis pathway genes (*GhNCED3a*, *GhNCED3c*, *GhAAO3*) and ROS-related genes was markedly downregulated (**Figures 7A–O**). These alterations collectively led to excessive ROS accumulation and exacerbated membrane damage, thereby conferring heightened salt sensitivity to the silenced plants.

This study elucidated the role of *GhABA2* under salt stress. The results indicate that *GhABA2* may contribute to enhanced salt tolerance by modulating ABA biosynthesis and coordinating responses associated with antioxidant activity and osmotic adjustment. These findings align with the well-established function of ABA in plant adaptation to abiotic stress. The present work advances the understanding of ABA-mediated pathways in cotton and offers new insights into the function of *ABA2* homologs in key economic crops. However, the precise molecular mechanisms through which *GhABA2* regulates the salt stress response in cotton remain to be fully elucidated and warrant further systematic investigation. Future research could employ CRISPR/Cas9-mediated gene knockout strategies to generate homozygous *GhABA2* mutants. Comparing phenotypic differences and physiological indicators between mutants and WT plants could clarify the specific role of *GhABA2* in stress response and growth development (Sohail et al., 2025). Notably, while this study demonstrated that constitutive overexpression of *GhABA2* driven by the 35S promoter enhanced salt tolerance, it also led to a reduction in thousand-seed weight, likely associated with elevated ABA levels. This observation may be interpreted as a potential adaptive trade-off between stress tolerance and growth, rather than a mere side effect. Under stress conditions, plants often reallocate resources from growth-oriented processes to defense mechanisms—a strategic reprioritization that enhances survival at the expense of biomass accumulation. In the context of this study, sustained ABA accumulation in overexpression lines may antagonize growth-promoting hormone signaling or directly suppress seed-filling processes (Cheng et al., 2014), reflecting such a trade-off (Skirycz and Inzé, 2010). It is also important to acknowledge the inherent limitations of using the constitutive CaMV 35S promoter in this study. Unlike the native promoter of *GhABA2*, which likely confers precise spatiotemporal expression patterns and stress-inducible regulation, the 35S promoter drives continuous and ubiquitous high-level expression across all tissues and developmental stages. This non-physiological expression pattern may have amplified certain phenotypes—particularly the growth suppression observed in overexpression lines—and could potentially distort the interpretation of GhABA2’s native function. Therefore, while our findings establish a role for *GhABA2* in salt tolerance, they may not fully recapitulate its natural regulatory context. To address this, future studies could optimize the expression pattern using tissue-specific promoters. A root-specific promoter could achieve local high expression of *GhABA2* at the key site of salt stress perception, improving root salt tolerance while minimizing negative impacts on shoot growth (You et al., 2013; Zhang and Liu, 2017; Tammi et al., 2023). Thereby enhancing stress resistance and reducing adverse effects on cotton agronomic traits to the greatest extent.

In practical applications, at the transcriptional regulatory level, it is essential to systematically verify the interaction between the *GhABA2* promoter and transcription factors by using yeast one-hybrid assays, electrophoretic mobility shift assays, dual-luciferase reporter gene assays, and ChIP-qPCR. Subsequently, functional analysis of the identified regulatory factors can be conducted using CRISPR/Cas9 and overexpression techniques. By monitoring changes in *GhABA2* expression and assessing phenotypic and physiological responses to ABA and abiotic stress, their regulatory roles in cotton stress resistance can be elucidated. Furthermore, investigating potential synergistic or antagonistic interactions will help refine the *GhABA2*-mediated transcriptional regulatory network (Wei et al., 2020; Arlt et al., 2023; Xie et al., 2023).

Furthermore, as an enzyme of the SDR family, *GhABA2* shares conserved motifs, but its members often exhibit different substrate specificities and significant functional diversity. The tetraploid nature of the cotton genome, the functional redundancy among homologous genes, and the transient nature of VIGS technology make it difficult to establish a clear link between enzymatic function and physiological phenotypes. It is therefore necessary to express and purify recombinant *GhABA2* protein for *in vitro* enzymatic activity assays and to construct stable *GhABA2* gene knockout mutants. Subsequently, ABA levels, stress response phenotypes, and growth indicators should be compared between wild-type and mutant plants. An *in situ* complementation assay should then be performed by reintroducing the wild-type *GhABA2* gene into the knockout background and observing the phenotypic rescue effect. Transcriptomic analysis of ABA biosynthesis-related genes in these materials can further verify their functional association.

This study identifies *GhABA2* as a positive regulator of salt tolerance in cotton, establishing its potential as a target for molecular breeding. Notably, a recent genome-wide association study identified a significant locus associated with climacteric ripening in melon, with *CmABA2* emerging as the candidate gene. *CmABA2* encodes a cytoplasmic short-chain dehydrogenase/reductase involved in ABA biosynthesis, offering a valuable genetic resource for melon breeding. Although the reported phenotypes differ (ripening regulation in melon versus salt tolerance in cotton), both genes share a conserved role in ABA biosynthesis, revealing a fundamental functional parallel. This evolutionary conservation underscores the broader relevance of our findings and suggests that *ABA2* homologs may serve as common regulatory nodes for maintaining ABA homeostasis across plant species, even when influencing distinct physiological traits. Future research will explore natural allelic variation of *GhABA2* within cotton germplasm resources. We hypothesize that high-expression alleles or haplotypes with enhanced function may exist in salt-tolerant accessions (Huang et al., 2012; Voss-Fels et al., 2019; Wang et al., 2021). By conducting genotype-phenotype association analysis in large cotton populations, we aim to identify superior *GhABA2* variants significantly correlated with enhanced salt tolerance. Such natural variation, combined with the functional insights provided here, could be utilized in marker-assisted selection to rapidly pyramid favorable alleles for developing new salt-tolerant cotton cultivars.

To summarize, *GhABA2* may as a key candidate gene for salt-tolerant cotton breeding, providing both a theoretical foundation and valuable genetic resources for improving cotton varieties in saline-alkali soils. This aligns with the urgent demand for salt-tolerant crop cultivars in the salinized cotton growing regions of Northwest China. Further in-depth research on *GhABA2* therefore holds significant theoretical value and promising application prospects.

## 5 Conclusions

In summary, this study identified *GhABA2* as a key positive regulator of salt stress tolerance in cotton. Functional validation in both cotton and *Arabidopsis* demonstrates that *GhABA2* enhances tolerance through coordinated activation of ABA biosynthesis and antioxidant defenses, leading to reduced oxidative damage and improved osmotic adjustment. These findings showed that *GhABA2* may as a promising target for molecular breeding aimed at improving salt tolerance in cotton and other crops.

## Data Availability

The datasets presented in this study can be found in online repositories. The names of the repository/repositories and accession number(s) can be found in the article/**Supplementary Material**.
