# Supplementary material for: Overexpression and silencing of the cotton GhABA2 gene reveal its role in salt stress tolerance
Source: Front Plant Sci. 2026 Apr 1;17:1803231. doi: 10.3389/fpls.2026.1803231 (PMC13079156; doi:10.3389/fpls.2026.1803231)
Supplement: Supplementary file 1 [file Supplementaryfile1.docx]

>Hibiscus trionum [GMI84923.1]

MATSSSNSLDSSLPTQRLAGKVALVTGGAAGIGEAIVRLFHKHGAKVCIVDVQDSLGLQVCQSLGDTPDI

CFFHCDVSKEEEVRCAVDFAVDKFGTLDIMVNNAGLSGPPCGDIRNYDLSEFEKVMDVNVKGVFLGMKHA

ARIMIPRGKGSIISMCSVSSVIGGLGPHAYTGSKHAVLGLNRSVAAELGKYGIRVNCVSPYAVATDLAFA

HLHEDERTEDVREGFRSFIGRNANLSGVDLTVEHVANAVLFLASDEAGYISGDNLMVDGGFTISNHALRVFR

>Corchorus olitorius [OMO75875.1]

MAASSCTVNSCGSSLPSQRLEGKVALVTGGASGIGESIVRLFHKHGAKVCIVDVQDNRGQQLCESLSDGS

NVSFFHCDVTIEEEVRAAVDFVVNEFGTLDIMVNNAGVSGPPHPDIRNYDLSDFEKVMDVNVKGVFLGMK

HAARVMIPNEKGSIISLCSVSSILGGIGPHGYTASKHAVLGLNKNVAAELGKYGIRVNCVSPYAVPTELS

FAHLHEDERTEEAIVGFRAFIGKNANLNGVDLTADHVANAVLFLASDEAGYISGDNLMVDGGFTSVNHSL

RVFR

>Ricinus communis [EEF35088.1]

MATTSSVASTLSCQRLLGKVALVTGGSTGIGESIVRLFHKHGAKVCLVDLEDNLGQNVCESLGGEPNICY

FHCDVTVEDEVQRAVEFTVDKFGTLDIMVNNAGLSGPPCPDIRYTELSDFQKVFDVNVKGTFIGMKHAAR

IMIPLNKGSIISLCSVASTIGGLGPHGYTGSKHAVLGLTRNVAAELGKHGIRVNCVSPYAVPTNLALAHL

HEDERTEDAMAGFKAFARKNANLQGVELTADDVANSVLFLASEESRYISGENLMIDGGFTNSNHSLRVFR

>Sinningia speciosa [URQ29604.1]

MALRGSGDSSLPTQRLLGRVALVTGGASGIGESIVRLFHKHGAKVCIADIQEDVGQRLCESLDGGHDVTF

CHCNVTIEDDVKHAVDFTVDKFGTLDIMVNNAGLSGPPCPDIRNFELSTFEQIFDVNVKGVFLGMKHAAR

IMIPAKKGSIISICSVASVIGGMGPHAYTGSKHAVLGLTKNVAAELGKHGIRVNCVSPYGIATGLALAHL

PEDERTEDALVGFRNFVGKNANLQGVELTADDIANAAVFLASDEARYISGDNLMVDGGFTSANHSLRVFR

>Parasponia andersonii [PON74388.1]

MSSTSAKEGISLSTQRLLGKVALVTGGATGIGESIVRLFHKHGAKVCLVDVQDNLGQHVCETLGEPRAFY

FQCDVTTEDDVCHAVDFTVDKFGTLDILVNNAGVSGSPCNDIRNADLAEFEKVFDINVKGAFLGMKHAAR

IMIPRKKGSIVSLCSVASALGGMGPHPYTSSKYALLGLTKNVAAELGLHGIRVNCVSPYAIATNLALAHL

PEEERTEDAWTAFRDFVGRNANLQGVELTVDDVANAVLFLASDESRYISGENLMVDGGFTSVTHSLRVFR

>Solanum tuberosum [NP_001275184.1]

MADTSLPIQRLLGKVALVTGGATGIGESIVRLFHKHGAKVCIADIRDEVGQHVCETLGNDQNVCFIHCDV

TVEADVSNAVDFTVQKFGTLDIMVNNAGLSGPPIRDIRDYELSVFENVLDVNLKGAFLGMKHAARIMIPL

KKGAIVSLCSVASAIGGIGPHGYAASKYAVLGLTQNVAAEMGKHGVRVNCVSPYAVATGLALAHLPEDEK

TDDAMEGFRDFVARNANLQGVELMANDVANAVLFLASDESRYISGHNLMVDGGFSCVNHSLRVFR

>Arachis hypogaea [AZM66048.1]

MSSSINNHHPQPSLPSQRLLGKVALVTGGACGIGESITRIFHIHGAKICIADIQDDIGKKVCESLGGEEN

VCFFHCDVASEDDVSRAIDFTVVKFGTVDIVVNNAGISGSPCPDIRHADLSEFEKVFNINVKGVFHGMKH

AARIMIPRKTGSIISLASVSSTTGGLGPHAYTGSKHAVLGLTKNVAAELGKYGIRVNCVSPYGVLTDLAL

AHLPEDERTEDAKTGFRDFVARNANLQGVEFTKDDVANAVLFLASDEAKYISGANLMLDGGFTSVNHSLK

VFRS

>Dorcoceras hygrometricum [KZV18922.1]

MVDFAAHCSSMAATGSDRSDLAAQRLLGRVALVTGGASGIGESIVQLFYKHGAKVCIADIQEDLGQRLCK

SLDGGISVTFCLCDVKVEADIQRAVDFTVDKFGSLDIMVNNAGLSGPPCPDIRDFDLSVFDHIFDVNVKG

VFLGMKHAARVMIPAKKGSIISTCSVASVIGGIGPHAYVGSKHAVLGLTKNVAAELGKHGIRVNCVSPYA

VATGLALAHLPEDERTEDALVGFRNFAGANANLQGVELTPQDVANAVVFLASDEARYISGANIMIDGGFT

SSNHSLRVFR

>Gossypium hirsutum [Ghi_A13G00706]

MATSSSNPIDSSLSSQRLVGKVALVTGGATGIGESIVRLFHKHGAKVCIVDVQDNLGLQVCQSLGNGPNVCFFHCDVTIEEQVRAAVDYAVEKFGTLDIM

VNNAGLSGPPYNDIRNYDLSDFEKVMNVNVKGVFLGMKHAARIMIPHEKGSIISTCSVSGVIGGLGPHAYTGSKHAVLGLTRNVASELGKYGIRVNCVSP

YAVATELAFAHLHEDERTEDVRTGFRAFIGKNANLNGVDLTVEHVANAVLFLASDDAGYISGDNLMVDGGFTSSNHSLRVFR

>Arabidopsis [AT1G52340.1]

MSTNTESSSYSSLPSQRLLGKVALITGGATGIGESIVRLFHKHGAKVCIVDLQDDLGGEVCKSLLRGESKETAFFIHGDVRVEDDISNAVDFAVKNFGTLDILINNAGLCGAPCPDIRNYSLSEFEMTFDVNVKGAFLSMKHAARVMIPEKKGSIVSLCSVGGVVGGVGPHSYVGSKHAVLGLTRSVAAELGQHGIRVNCVSPYAVATKLALAHLPEEERTEDAFVGFRNFAAANANLKGVELTVDDVANAVLFLASDDSRYISGDNLMIDGGFTCTNHSFKVFR

>Oryza sativa [LOC_Os03g59610.1]

MSAAAAAAASSPAPRLESKVALVTGGASGIGEAIVRLFREHGAKVCIADIQDEAGQKLRDSLGGDQDVLFVHCDVSVEEDVARAVDATAEKFGTLDIMVNNAGFTGQKITDIRNIDFSEVRKVIDINLVGVFHGMKHAARIMIPNKKGSIISLGSVSSVIGGLGPHSYTATKHAVVGLTKNVAGELGKHGIRVNCVSPYAVPTALSMPYLPQGERKDDALKDFFAFVGGEANLKGVDLLPKDVAQAVLYLASDEARYISALNLMVDGGFTSVNHNLRAFED

>Zea mays [ZmPHB47.05G018800.1]

MAAAGSSPSFSSSSKRLEGKVALVTGGATGIGEAIVRLFMEHGAKVCIADIQDEAGQQLRDALGGDAQGAMFVHCDVTSEEDVSRAVDAAAERFGALDVMVNNAGVTGTKVTDIRNVDFAEARRVLDVNVHGVFLGMKHAARAMIPRKRGSIVSLASVASAIGGTGPHVYTASKHAVVGLTKSVAAELGRHGVRVNCVSPYAVPTALSMPHLPQGARADDALKDFLAFVGGEANLKGVDAMPEDVAQAVLYLASDEARYVSAVNLMVDGGFTAVNNNLRAFED

>L.usitatissimum [Lus10016354]

MASSSSSPVPSPATQSQRLSGKVALITGGASGIGESIARLFYKHGAKLCIVDLQDNLGDRLCQSLGGGPNGVYYLHCDVTNEEHISRAVDFAAEKFGTIDILVNNAGITGPPCSDIRDAKLSDYELVFDVNVKGTFLGMKHAARVMIPSSKRGSIINLCSVAGNMGGLGPHTYTGSKHAVLGLTRSVAAELGKYGIRVNCVSPYAVPTGLALAHLPEDERTEDAMAGFRAFAEKNANLNGVGLEPEDVANAVLFLASDEARYISGDNLMVDGGLTCTNHSFRVFR
