# Supplementary material for: Overexpression and silencing of the cotton GhABA2 gene reveal its role in salt stress tolerance
Source: Front Plant Sci. 2026 Apr 1;17:1803231. doi: 10.3389/fpls.2026.1803231 (PMC13079156; doi:10.3389/fpls.2026.1803231)
Supplement: Supplementary file 3 [file Supplementaryfile3.docx]

**Supplementary Information**

**Measurement of endogenous ABA levels**

To determine the ABA content in cotton and Arabidopsis thaliana tissues, a commercial competitive Enzyme-Linked Immunosorbent Assay (ELISA) kit (Jiangsu Jingmei Biological Technology Co., Ltd., Yancheng,, China) was used according to the manufacturer's instructions.

Sample Extraction Details: Cotton and Arabidopsis leaf samples were ground to a fine powder in liquid nitrogen. An accurately weighed amount of the powder was homogenized in ice-cold phosphate-buffered saline (PBS, 0.01 M, pH 7.4). The ratio of fresh weight (FW) to PBS volume was [e.g., 1:9] (i.e., 0.1 g tissue in 1 mL PBS). The homogenate was thoroughly ground on ice and then centrifuged at 5000 rmp for 10 minutes at 4℃. The supernatant was carefully collected for subsequent analysis. The entire extraction process was performed on ice and protected from light. Samples were either assayed immediately or stored at -80℃ until use, avoiding repeated freeze-thaw cycles. According to the kit's precautions, no sodium azide (NaN3) was used in any buffers to avoid inhibition of HRP activity.

Assay Procedure and Standard Curve: The assay was performed strictly following the manual. Standard wells and sample wells were set up. For sample wells, 10 μL of the test sample and 40 μL of Sample Diluent were added (resulting in a 5-fold dilution of the sample). Subsequently, 50 μL of HRP-conjugated antigen was added to each well and incubated for 60 minutes at 37°C. After washing five times with wash buffer, chromogen solutions were added for color development. The optical density (OD) was measured at 450 nm. A standard curve was generated using the standard concentrations (0, 6.25, 12.5, 25, 50, 100 ng/mL) and their corresponding OD values.

Method Validation: To evaluate the accuracy of the method and potential matrix effects, a spike recovery test was performed. The results showed that after spiking cotton and *Arabidopsis* thaliana leaf samples with a standard concentration of 12.5 ng/mL, the detected ABA concentrations in the spiked samples were 20.9827 ng/mL and 18.196938 ng/mL, respectively. The ABA concentrations in the corresponding unspiked samples were 9.862923 ng/mL and 7.86796 ng/mL, respectively. The recovery rates (R) were calculated using the following formula:

R(%)=（C加标−C本底）/C加入×100%

C spiked:Measured concentration of the spiked sample.

C background:Measured concentration of the background (unspiked) sample.

C added:Concentration of the standard added.

The final recovery rates were determined to be 88.18% and 82.63% for cotton and Arabidopsis, respectively, both falling within the acceptable range of 80%–120%. These results indicate that the method possesses satisfactory accuracy and that no significant matrix interference was observed in the two plant matrices tested in this study.

Calculation and Data Presentation:

The sample well concentration calculated from the standard curve was multiplied by the sample dilution factor (5-fold for *Arabidopsis* and 10-fold for cotton). The final ABA content was expressed as nanograms per gram of fresh weight (ng/mg FW) and calculated using the following formula:

ABA content (ng/g FW)=(C×V)/W

where C is the sample concentration (ng/mL) interpolated from the standard curve, V is the total volume of the extract (mL), and W is the fresh weight of the sample (g). All samples were measured in three technical replicates, and the results are presented as mean ± standard error.


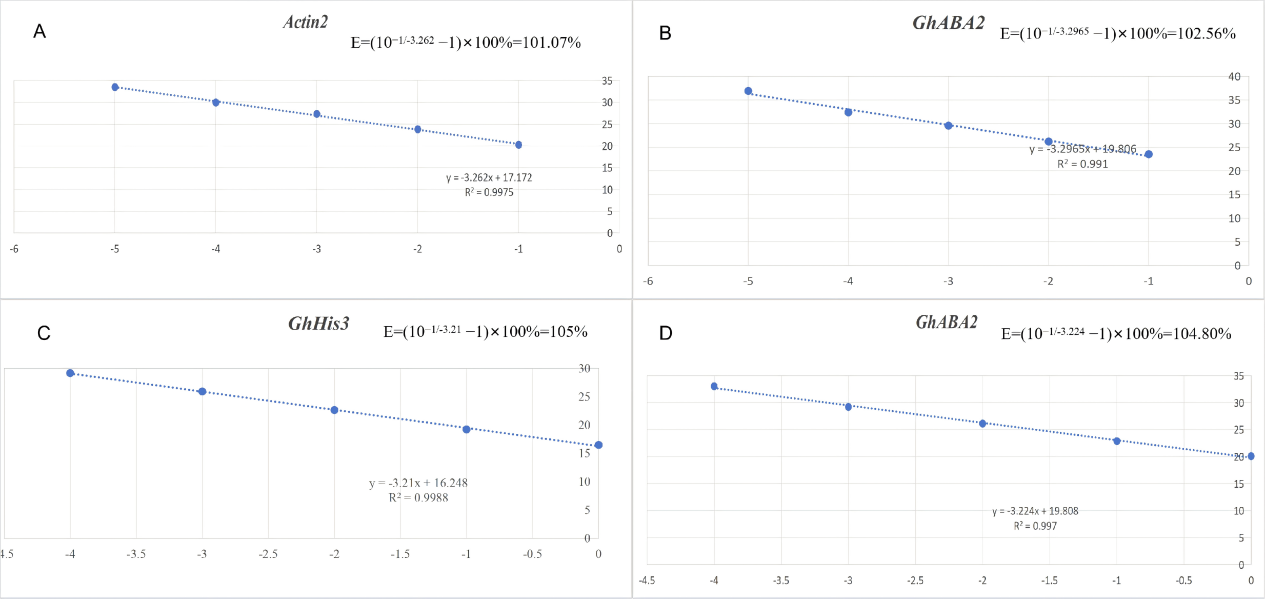


Figures.S1 Primer Amplification Efficiency

1. Amplification efficiency curve of the *Actin2* qRT-PCR primers; (B) Amplification efficiency curve of the *GhABA2* qRT-PCR primers in Arabidopsis; (C) Amplification efficiency curve of the *GhHis3* qRT-PCR primers; (D) Amplification efficiency curve of the *GhABA2* qRT-PCR primers in cotton.


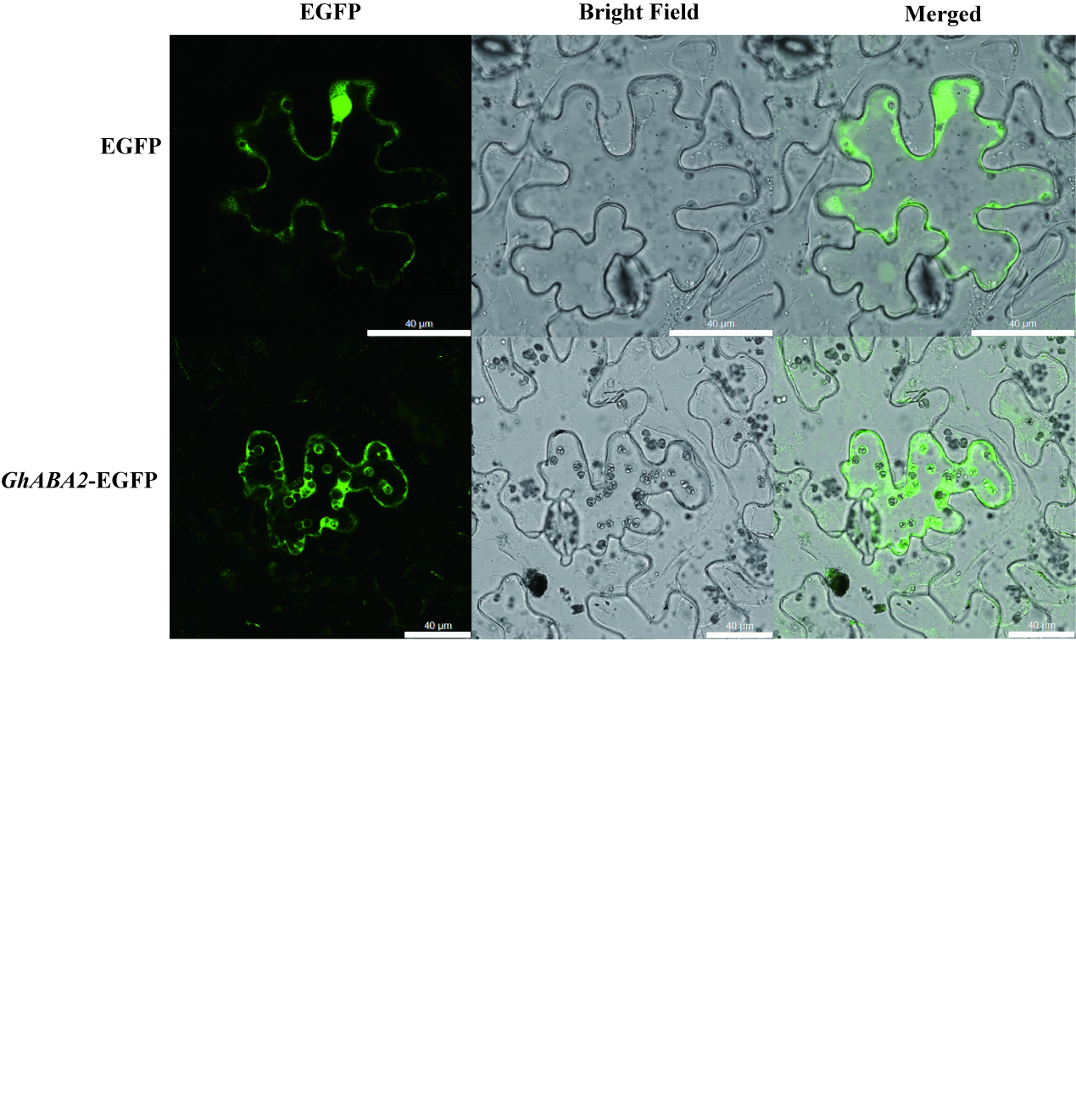


Figures.S2 Subcellular localization of GhABA2


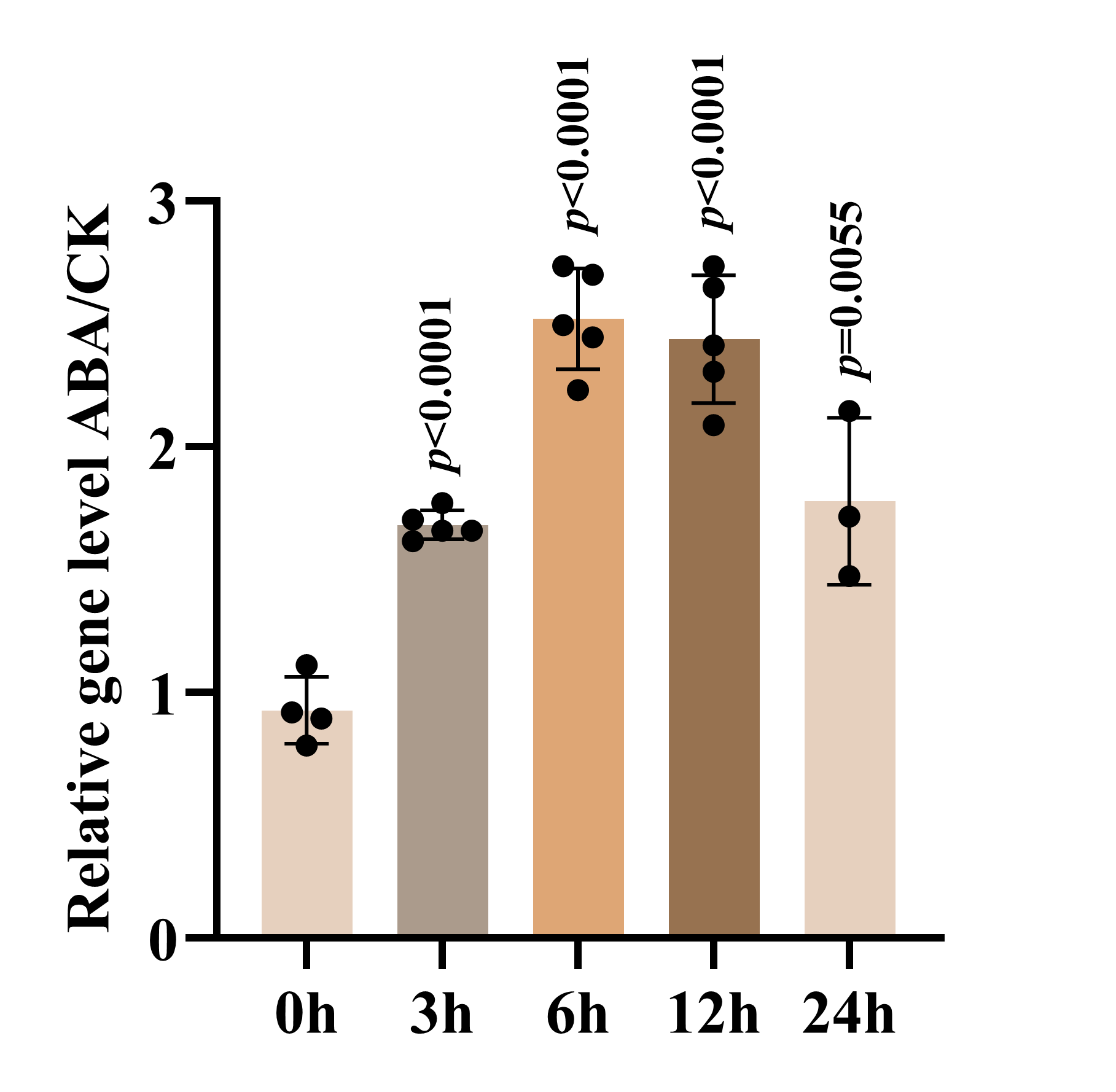


Figures.S3 Expression of the *GhABA2* gene under ABA treatment.。

This figure examines the expression of the *GhABA2* gene under ABA treatment conditions. The X-axis represents the duration of ABA treatment, while the Y-axis shows the ratio of *GhABA2* gene expression under ABA treatment to its expression under control (CK) conditions at the corresponding time points.


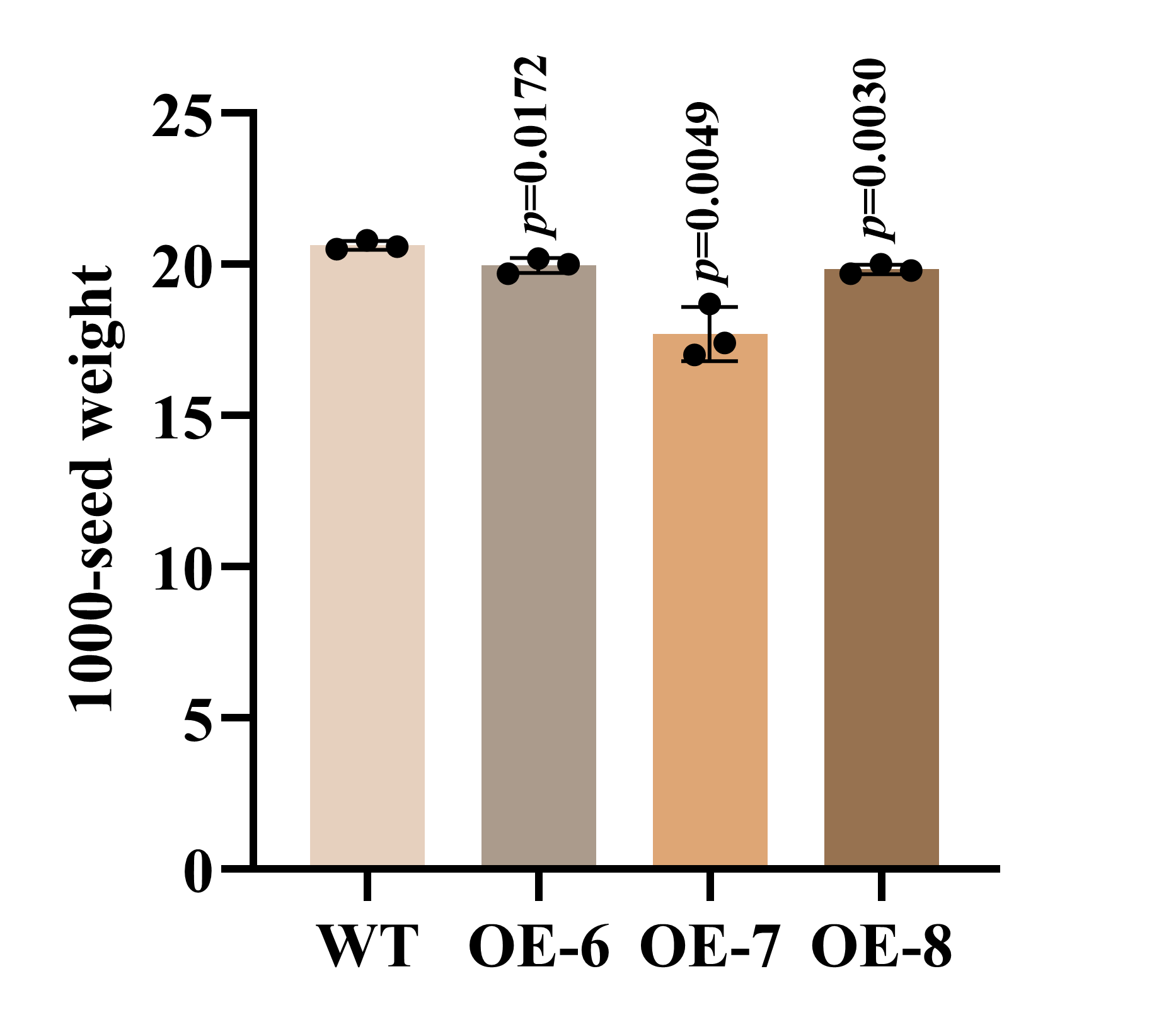


Figures.S4 the 1000-seed weight of *Arabidopsis* seeds overexpressing *GhABA2*


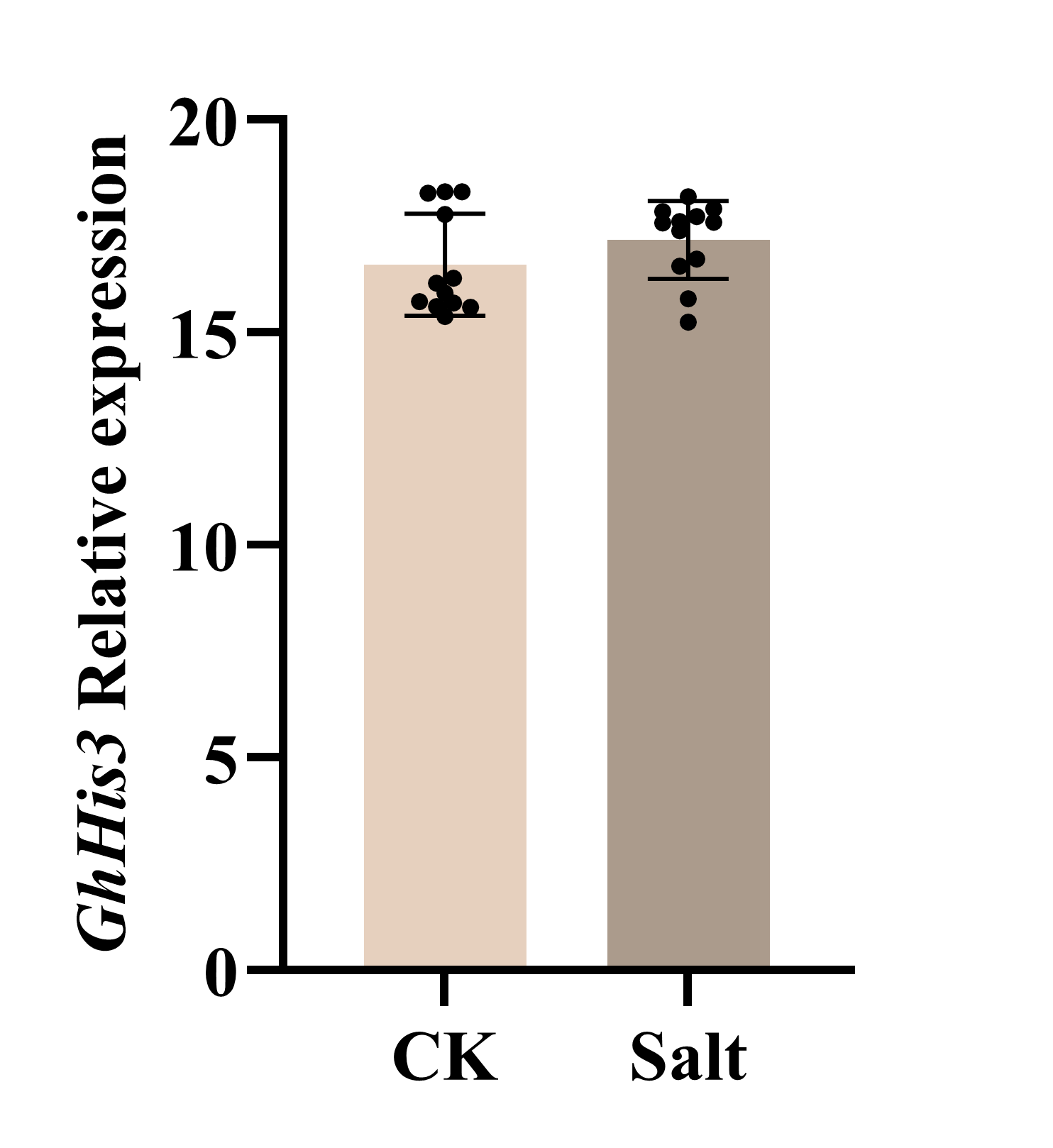

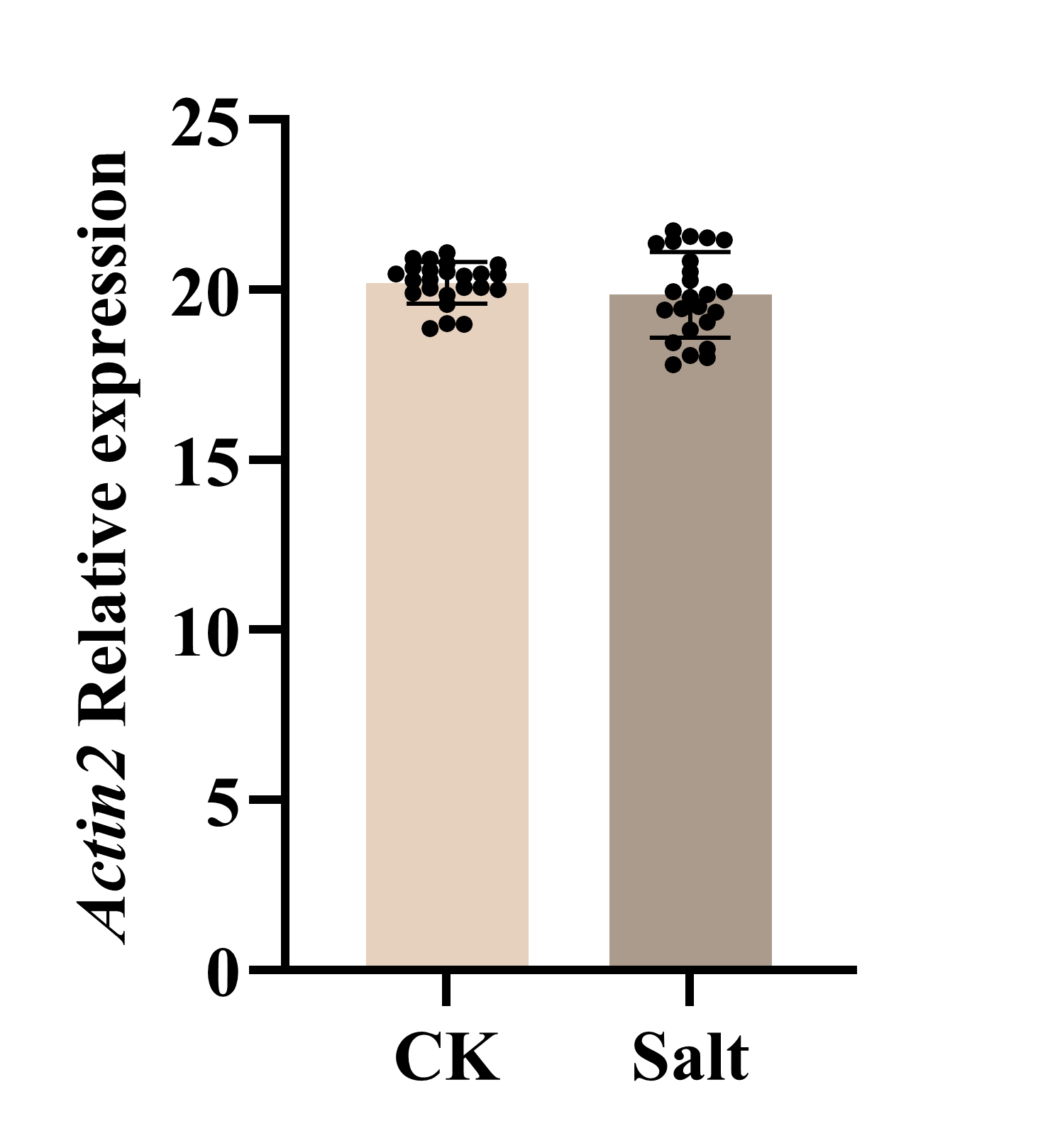


Figures.S5. Expression stability validation of reference genes *GhHis3* and *Actin2* under salt stress.

A


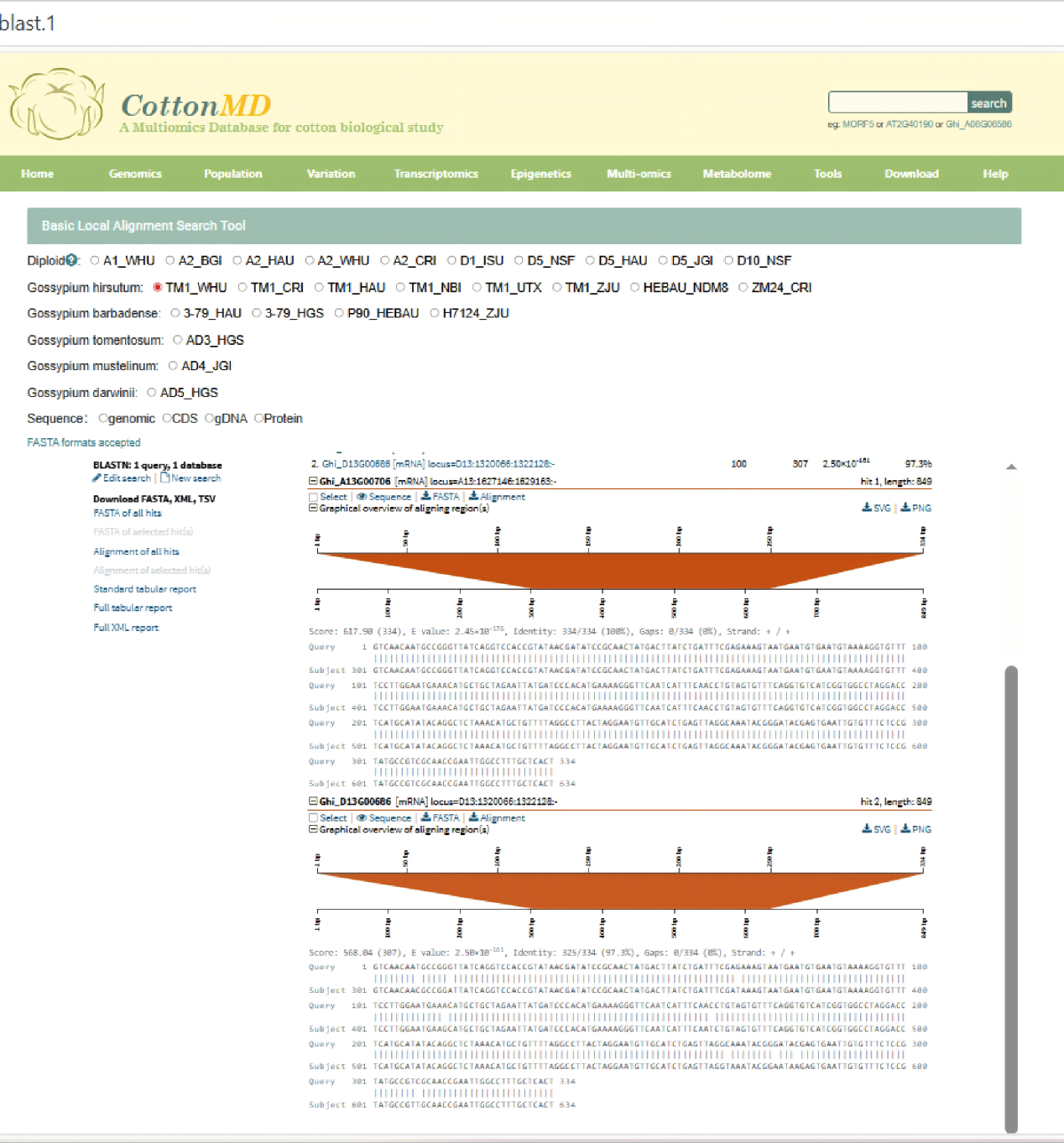


B


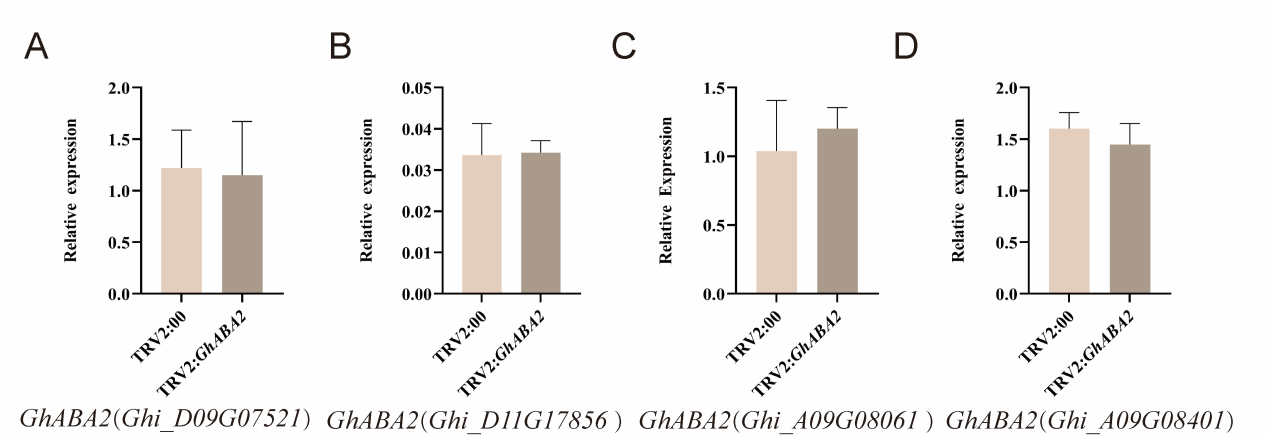


c

D


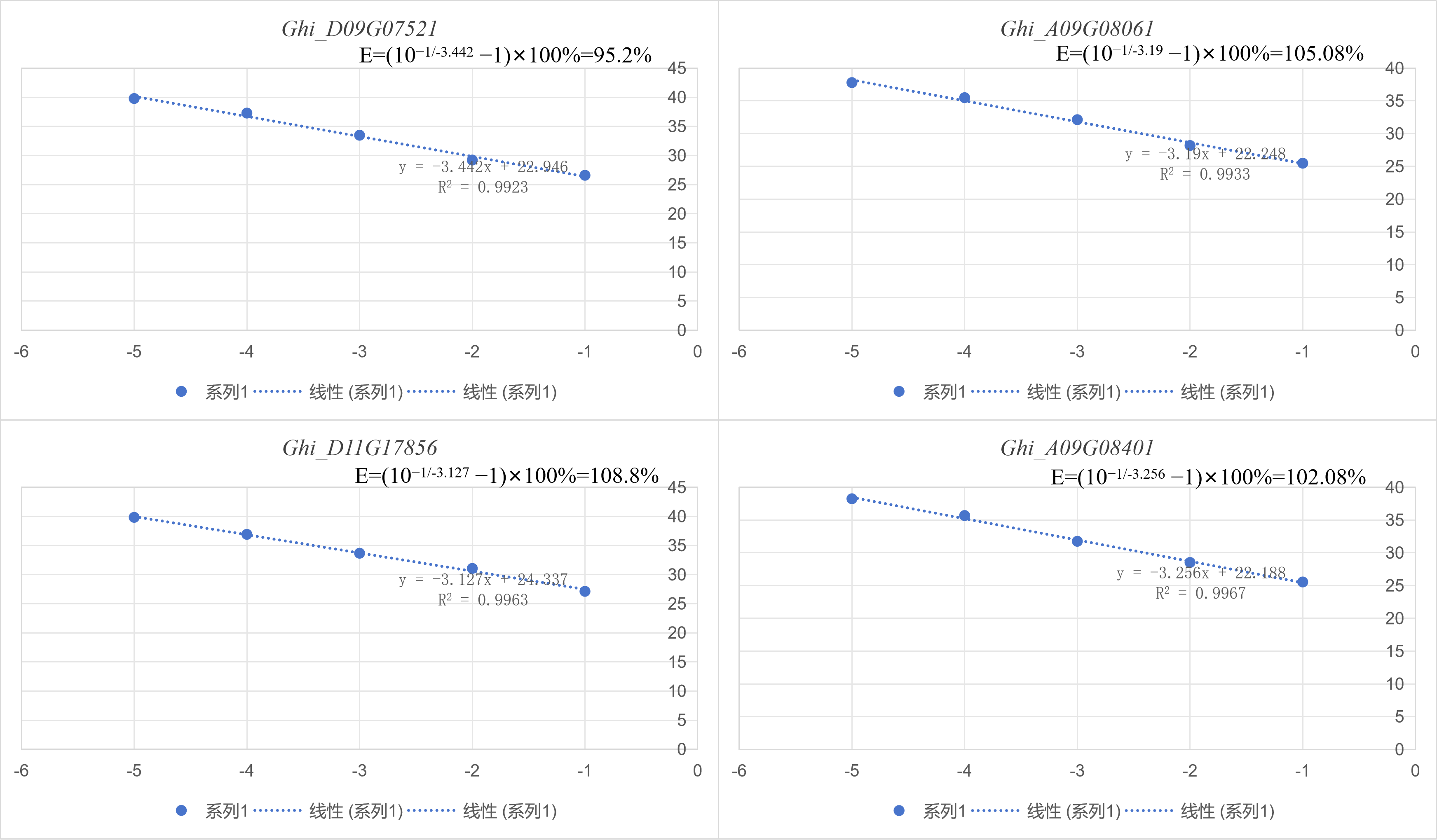


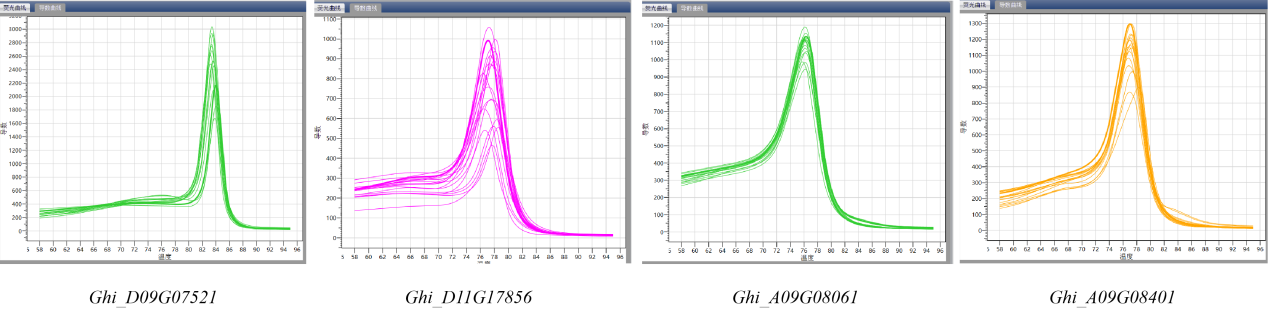


Figures.S6 Specificity analysis of the *GhABA2* TRV2-specific fragment.

(A) Sequence alignment of the *GhABA2* TRV2-specific fragment; (B) Expression levels of *GhABA2* homologous genes in the TRV2:*GhABA2*-silenced plants; (C) Results of sequence alignment between TRV2-*GhABA2* and its homologous genes; (D) Amplification efficiency and product specificity of primers for *GhABA2* homologous genes.


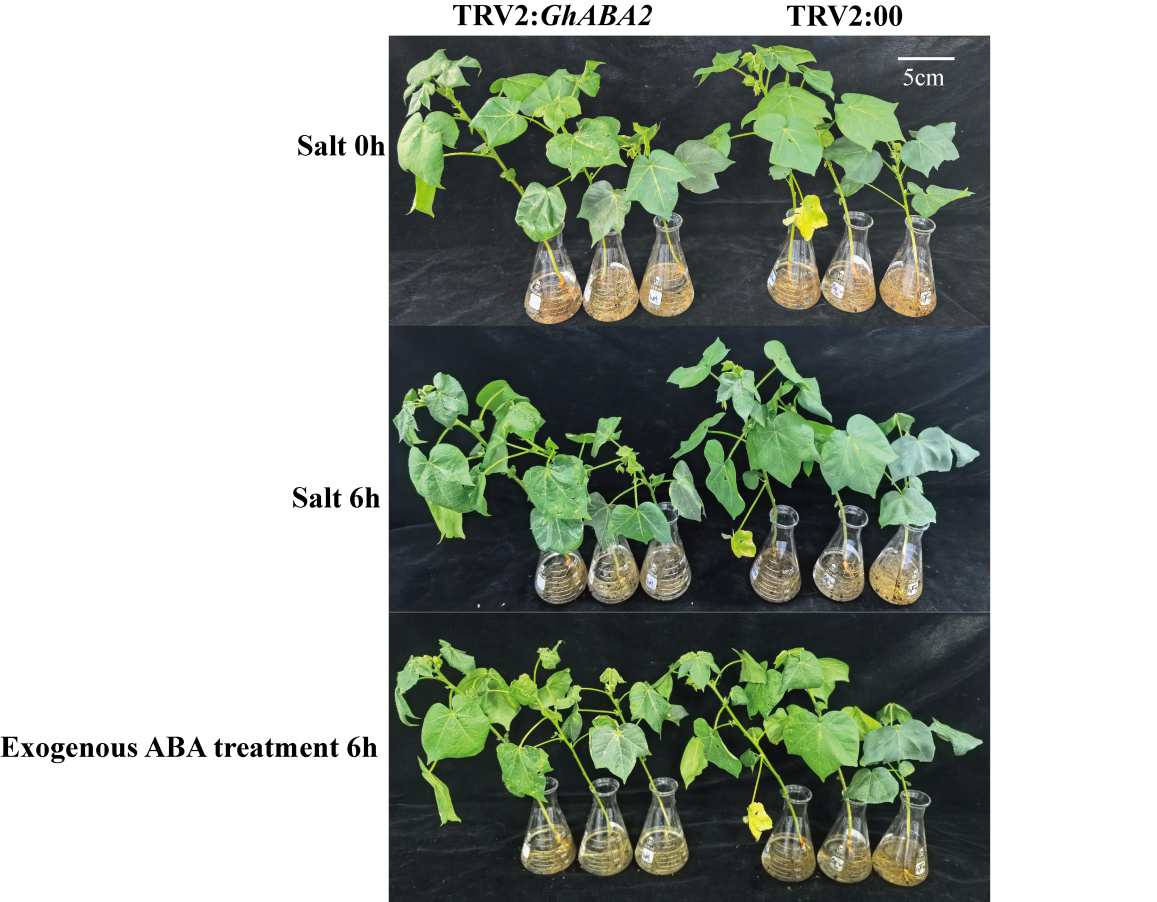


Figures.S7 Phenotypic rescue of *GhABA2*-silenced cotton plants by exogenous ABA under salt stress

Table S1. Bioinformatic Analysis Software

| **Software** | **Website** | **Functionality** |
| --- | --- | --- |
| Prot Param | http://web.expasy.org/cgi-bin/protparam/protparam | Physicochemical property analysis |
| NetPhos 3.1 | http://www.cbs.dtu.dk/services/NetPhos | Phosphorylation site analysis |
| Prot Scale | http://www.expasy.org/tools/protscale.html | Hydrophobicity/hydrophilicity analysis |
| PSORT | http://www.genscript.com/cgi-bin/tools/psort2.pl | Subcellular localization analysis |
| SOPMA | https://npsa-prabi.ibcp.fr/cgi-bin/secpred_sopma.pl | Secondary structure analysis |
| SWISS-MODEL | http://swissmodel.expasy.org | Tertiary structure analysis |
| DNAMAN | - | Multiple sequence alignment |
| MEGA11.0.13 | - | Phylogenetic tree construction |
| Plantcare | https://bioinformatics.psb.ugent.be | Cis-acting element analysis |

Table S2. Prediction of cis-acting elements in the *GhABA2* promoter

| **Element** | **Number** | **Function** |
| --- | --- | --- |
| TGACG-motif | 1 | cis-acting regulatory element involved in the MeJA-responsiveness |
| TC-rich repeats | 2 | cis-acting element involved in defense and stress responsiveness |
| ABRE | 1 | cis-acting element involved in the abscisic acid responsiveness |
| ARE | 1 | cis-acting regulatory element essential for the anaerobic induction |
| GA-motif | 1 | part of a light responsive element |
| GT1-motif | 3 | light responsive element |
| Box 4 | 4 | part of a conserved DNA module involved in light responsiveness |
| CGTCA-motif | 1 | cis-acting regulatory element involved in the MeJA-responsiveness |
| G-box | 1 | cis-acting regulatory element involved in light responsiveness |
| O2-site | 1 | cis-acting regulatory element involved in zein metabolism regulation |

GhABA2 VIGS-specific sequence

ATAGAAGAACAAGTTCGGGCCGCGGTCGACTACGCAGTTGAAAAGTTTGGTACACTCGATATCATG

GTCAACAATGCCGGGTTATCAGGTCCACCGTATAACGATATCCGCAACTATGACTTATCTGATTTCGAGAAAGTAATGAATGTGAATGTAAAAGGTGTTT

TCCTTGGAATGAAACATGCTGCTAGAATTATGATCCCACATGAAAAGGGTTCAATCATTTCAACCTGTAGTGTTTCAGGTGTCATCGGTGGCCTAGGACC

TCATGCATATACAGGCTCTAAACATGCTGTTTTAGGCCTTACTAGGAATGTTGCATCTGAGTTAGGCAAATACGGGATACGAGTGAATTGTGTTTCTCCG

TATGCCGTCGCAACCGAATTGGCCTTTGCTCACT
